# Siblings of Persons with Disabilities: A Systematic Integrative Review of the Empirical Literature

**DOI:** 10.1007/s10567-024-00502-6

**Published:** 2024-10-16

**Authors:** Annalisa Levante, Chiara Martis, Cristina Maria Del Prete, Paola Martino, Patrizia Primiceri, Flavia Lecciso

**Affiliations:** 1 Department of Human and Social Sciences, University of Salento, Via di Valesio, 73100 Lecce, Italy; 2 Lab of Applied Psychology, Department of Human and Social Sciences, University of Salento, Via di Valesio, 73100 Lecce, Italy; 3 District of Rehabilitation, Local Health Service, 73100, P.zza Bottazzi, Lecce, Italy; 4 Office for Inclusion of Individuals with Disability, University of Salento, Via di Valesio, 73100 Lecce, Italy

**Keywords:** Sibling, Disability, Systematic integrative review, Parentification, Sibling relationship, Emotional/behavioral adjustment, Well-being

## Abstract

The systematic review aimed to systematize the empirical literature on the psychological impact of disability on the siblings of persons with disabilities, to inform research and provide clinical recommendations. Two research questions addressed the review: (1) *What are the main psychological constructs investigated in siblings of persons with disability*? (2) *What is the main role of each psychological construct in siblings of persons with disability experience*? The electronic search was conducted in 7 databases and the PRISMA diagram was used. The inclusion criteria were: Papers published in English and in peer-reviewed journals; papers published between January 2014 and June 2024; qualitative, quantitative, and mixed studies; and papers on the psychological impact of disabilities and/or chronic illnesses on the experience of siblings of persons with disabilities. The standardized Mixed Method Appraisal Tool protocol was used to appraise the methodological quality of the studies. To summarize the findings, a narrative approach was adopted. A total of 60 studies have been reviewed. According to the methodological quality appraisal of studies, most of them reported a high (*n* = 45) and medium (*n* = 15) quality. They involved 10,146 participants. Findings revealed that sibling relationships, sibling-focused parentification, and emotional/behavioral adjustment are the main psychological constructs investigated by existing literature. Few studies focused on siblings’ well-being. Studies exploring more than a psychological construct were included as a hybrid. Only one study examined the siblings’ psychological experience during the COVID-19 pandemic. The quality of the sibling relationship ranged from good to poor; the caregiver is the main role played by siblings of persons with disabilities; anxiety, depression, and aggressive behaviors are the main emotional/behavioral symptoms revealed. Most studies (*n* = 39) recruited participants with brothers or sisters with mental disorders. The findings of this systematic review may play a role in the clinical field, as they might help to design gender- and age-specific intervention programs.

## Introduction

The Bioecological Systems Theory (Bronfenbrenner, 1977;1979; Tudge et al., 2009) holds that the child’s development results from a complex system of relationships that are influenced by the environment. According to Minuchin’s Family System Theory, family members affect each other, thus when a child is diagnosed with a disability and/or a chronic illness, they are significantly touched (Barnett et al., 2003). Because mothers often are the primary caregivers of children, most of the research carried out on the reaction to the diagnosis of disability in the family (Marvin & Pianta, 1992; Sher-Censor et al., 2022) devoted attention to them. Results highlighted high levels of distress (Al-Kandari & Al-Qashan, 2010; Lecciso et al., 2013a, 2013b; Lecciso et al., 2013a, 2013b; Sato et al., 2015) and depressive symptoms (Dillon-Wallace et al., 2014; Ingersoll & Hambrick, 2011; Rashedi et al., 2013). On fathers, studies reported that they devote more time and energy to work (Keller & Honig, 2004; Zanobini et al., 2002) and show lower depressive symptoms than mothers (McStay et al., 2014; Vasilopoulou & Nisbet, 2016). Over the last few years, a substantial number of studies have been carried out on typically developing siblings (henceforth siblings), whose functioning may be significantly altered by growing up with a brother or sister with a disability. Nevertheless, findings on this topic are mixed. Most studies (Giallo & Gavidia-Payne, 2006; Hastings, 2003; O’Neill & Murray, 2016; Petalas et al., 2009; Ross & Cuskelly, 2009; Rossetti & Hall, 2015; Sharpe & Rossiter, 2002; Stoneman, 2005; Williams et al., 2010) revealed that the brother’s or sister’s disability negatively affected the functioning of the sibling, causing high levels of anxiety and depression, social withdrawal, conduct disorders, and other behavioral problems. Other studies (Kaminsky & Dewey, 2001; Moyson & Roeyers, 2012; Opperman & Alant, 2003; Rossetti & Hall, 2015) showed that having a brother’ or sister’s disability increases empathy and psychosocial development, self-perceived competence, high-quality of life, and improved family cohesion in siblings of persons with disabilities. Finally, albeit they are few (Cuskelly & Gunn, 2003; Hallion et al., 2018), studies have found no difference between siblings of persons with disabilities and siblings of typically developing individuals.

Considering siblings the family members might become the main caregivers for their brother or sister with a disability, we conceived this systematic integrative review to provide an overview of empirical existing studies investigating the psychological impact of disability on this vulnerable population. Previous systematic reviews have been conceived to address this purpose; nevertheless, they examined the impact of disability related to a specific psychological construct, that is well-being (Schamong et al., 2021), parentification (Eun Lee & Burke, 2018), psychological adjustment (Rochefort et al., 2023), empathy and prosocial behaviors (Long et al., 2018; Orm et al., 2021), and general psychopathology (Ma et al., 2015). Overall, albeit high levels of empathy and prosocial behaviors have been developed by growing with a brother or sister with disability (Orm, 2021), the results of these reviews outline a detrimental psychological effect of the disability on the sibling’s experience. Findings highlight that caregiver is the main role served by the siblings of persons with disabilities (Eun Lee & Burke, 2018) and that low well-being and quality of life is experienced (Schamong et al., 2021). In addition, externalizing (e.g., self-aggression) and internalizing (e.g., anxiety) symptoms have been retrieved (Long, 2018; Ma, 2015). Similarly, dysfunctional family relationships consisting of poor parental support (Rochefort et al., 2023) have been reported by siblings.

Additionally, these reviews considered studies on siblings of persons with a specific disability. For instance, neurodevelopmental disorders (Rochefort et al., 2023; Leedham et al., 2020; Orm et al., 2021; Thomas et al., 2015; Watson et al., 2021; Eun Lee & Burke, 2018) or mental health problems (Ma et al., 2015), chronic illnesses (e.g., asthma, and brain injury; Incledon et al., 2015; Knecht et al., 2015), cancer (Long et al., 2018; Yang et al., 2016), congenital heart disease (Parker et al., 2020; Schamong et al., 2021).

Based on the purposes of previous reviews and the target population whom they devoted attention as well, the current systematic review aims at synthesizing the existing literature on what psychological constructs have been explored on siblings of persons with disability. In addition, the current systematic review aimed at covering the last 10-year period to help scholars and health professionals in designing studies and intervention programs as well.

Based on this rationale, following the Population Exposure Outcome (PEO) format, two research questions (RQs) were formulated:

RQ1: *What are the main psychological constructs investigated in siblings of persons with disability?*

RQ2: *What is the main role of each psychological construct in siblings of persons with disabilities experience?*

Because the reviewed studies showed great heterogeneity in the way they measured the psychological constructs and reported results, an integrative narrative approach (Whittemore & Knafl, 2005) was adequate to synthetize the findings.

## Review Method

### Search Strategy

The current systematic review is based on the structure proposed by a published protocol (Levante et al., 2019). In extracting studies, the updated Preferred Reporting Items for Systematic Review and Meta-Analysis (PRISMA) diagram was applied (Page et al., 2021). The electronic search was carried out in 7 databases (i.e., Scopus, MEDLINE, PsychInfo, Cumulative Index to Nursing and Allied Health Literature, ERIC, PubMed, and Web of Sciences) covering the decade 2014–2024.

Table 1 shows the keywords and their MeSH terms selected by the PEO protocol. In each database, all keywords were combined using the Boolean operators “AND” & “OR.”

**Table 1:** Search strategy using Boolean operators (OR & AND) according to the PEO protocol

	P—Population	E—Exposure	O—Outcomes
Search strategy using Boolean Operators (AND & OR)	Siblings of persons with disabilities and/or chronic illness	Presence in the family of a child with disabilities and/or chronic illness	Impact of the brother/sister disability and/or chronic illness on psychological functioning of siblings
	“sib” OR “sibling” OR “healthy sibling” OR “sibling without disability*” AND	“disabilit*” OR “disable” OR “chronic illness” OR “people with disability*” AND	“psych* impact” OR “mental health” OR “psych* functioning”

^a^The Note reported the long name of the measure and the reference

A set of pre-defined inclusion and exclusion criteria were tabulated (Table 2).

**Table 2:** Inclusion and exclusion criteria

Inclusion criteria	Exclusion criteria
(a) papers published between 2014 and 2024 (b) papers published in peer-reviewed and indexed journals (c) papers are written in English (d) papers focused on the psychological impact of disabilities and/or chronic illness on the functioning of siblings of persons with disabilities (e) qualitative, quantitative, and mixed studies	(a) papers focused on non-psychological constructs (i.e., medical) (b) systematic, scoping, integrative, and narrative reviews (c) validation studies on measures evaluating psychological constructs in siblings of persons with disabilities (d) papers investigating the experience of siblings of persons with disabilities examining the intervention programs’ effectiveness (e) paper drawing attention to the impact of the disabilities and/or chronic illness on other family members (f) papers based on parent-reported evaluation of the experience of the siblings of persons with disabilities

### Selection of the Studies

**Fig. 1:**
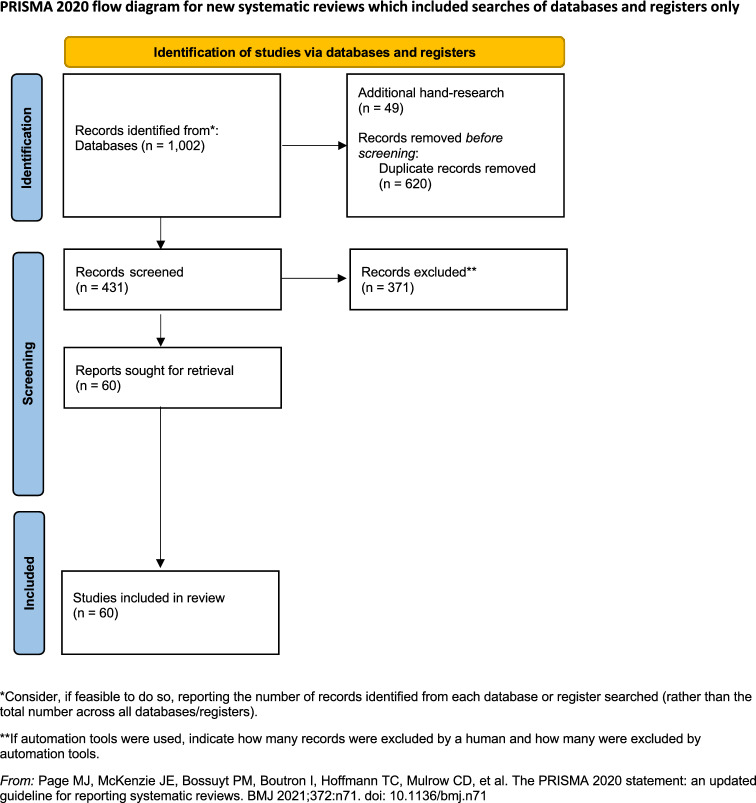
PRISMA flow diagram

As for the content of the studies, the Population Intervention Comparison Outcome Study (PICOS) protocol (Bowling & Ebrahim, 2005) was used: Participants: siblings of any age with a brother or sister with disabilities; Intervention: empirical studies assessing psychological constructs impacting the disabilities and/or chronic illness of the brother or sister; Comparison: differences according to the siblings’ gender evaluated in the reviewed studies; Outcomes: any psychological constructs**; S**tudy: quantitative; qualitative; mixed. Figure 1 shows the PRISMA (Page et al., 2021) diagram.

According to the first step of the PRISMA diagram (i.e., Identification), in each database, we searched papers in which our keywords appeared in either the paper’ title, abstract, subject heading, or keywords list. For each keyword combination, all records were tabulated in a.csv spreadsheet and a total of 1,002 records were alphabetically ordered. A second-hand search was performed to incorporate 49 records according to the reference lists of reviewed studies.

Through an automatic tool included in the.csv spreadsheet, duplicates (n = 620) were removed. A total of 431 papers were screened by two authors (AL & CM) for inclusion. Before the Screening step, the inter-rater agreement was calculated on a set of 50 papers, randomly chosen. They were independently screened by the two authors (AL & CM), and any disagreements were arbitrated by a third author (FL). The inter-rater agreement was good (Cohen’s *K* = 0.93).

A total of 371 papers were excluded according to the pre-defined criteria and the final number of included papers was 60 (marked with * in the references list) (Avieli et al., 2019; Beffel & Nuttall, 2020; Bhattashali et al., 2018; Braconnier et al., 2018; Brady et al., 2019; Brolin et al., 2024; Cebula et al., 2019; Chiu, 2022; Correia & Seabra-Santos, 2022; Corsano et al., 2017; Cuskelly, 2016; Dorsman et al., 2023; Floyd et al., 2016; Guidotti et al., 2021; Hallion et al., 2018; Hanvey et al., 2022; Hayden et al., 2023; Hemati Alamdarloo et al., 2021; Jones et al., 2019; Kale & Siğirtmaç, 2021; Kulisch et al., 2024; Koukouriki & Soulis, 2020; Lahaije et al., 2023; Lashewicz, 2018; Le Boudec et al., 2021; Eun Lee et al., 2018, 2023; Eun Lee et al., 2019a, 2019b; Eun Lee et al., 2019a, 2019b; Eun Lee et al., 2020; Levante et al., 2023a, 2023b, 2023c; Milevsky & Singer, 2022; Niedbalski, 2023; Noonan et al., 2018; Nuttall et al., 2018; O’Neill & Murray, 2016; Orm et al., 2022; Paul et al., 2022; Perenc & Pęczkowski, 2018; Redquest et al., 2020, 2021; Rossetti et al., 2018, 2020; Shivers, 2019a, 2019b; Shivers et al., 2019; Shivers & Kozimor, 2017; Shojaee et al., 2020; Siman-Tov & Adi Sharabi, 2023; Stock et al., 2016; Tomeny et al., 2016a, 2016b; Tomeny et al., 2016a, 2016b, 2017a, 2017b; Tomeny et al., 2017a, 2017b; Travers et al., 2020; Tsai et al., 2016; Tyerman et al., 2019; Yacoub et al., 2018; Yaldız et al., 2021; Zaidman-Zait et al., 2020).

### Methodological Quality Appraisal of Included Studies

The methodological quality appraisal of the included papers has been evaluated using the updated standardized protocol Mixed Method Appraisal Tool (MMAT; Hong et al., 2018). The protocol appraises the studies across five main categories: qualitative research, randomized controlled trials, non-randomized studies, quantitative descriptive studies, and mixed methods research. A set of five questions has been designed for each study category and response options for each question ranged on a 3-point Likert scale (1 = “yes,” 0 = “no,” and 0 = “can’t tell”). The MMAT authors provide a spreadsheet in which the reviewers report the main study information (i.e., reference ID, first author, year of publication, and full citation). Afterward, the reviewers fill out two screening questions (i.e., “*Are the research questions clearly stated*?” and “*Do the data collected address the research questions*?”). Whether the answer to these screening questions is “no” or “can’t tell,” the study is excluded. In contrast, if “yes” answers are provided, the reviewers complete the five relevant questions to appraise the methodological quality of the study. The inter-rater agreement is calculated on 20% of the included papers. For the current systematic review, the inter-rater agreement is excellent (Cohen’s κ = 0.95). The MMAT developers do not provide a cut-off to categorize the scores; nevertheless, they suggest using a conservative approach. Similar to another review (Levante et al., 2023a, 2023b, 2023c), the quality appraisal is low if the study reaches 1 or 2 “yes” answers, medium if it scores 3 “yes,” and high if the study reaches 4 or 5 “yes” answers.

## Results

Table 3 summarizes the main information provided in the reviewed studies which are clustered according to the psychological constructs examined and the methodological quality appraisal of each study.

**Table 3:** Overview of all included studies’ descriptive characteristics

Construct	Author(s), year	Country	Study design (cross-sectional *vs* longitudinal; quantitative *vs* qualitative *vs* mixed)	Participants (sample size; gender distribution; mean age and standard deviation; age range)	Type of disabilities and/or chronic illness considered	Measure(s) (in bracket the psychological construct evaluated)^a^	Main Findings	Quality Appraisal
*Sibling Relationship*								
1	Paul et al., 2022	Global South (El Salvador, Guatemala, Puerto Rico, Bangladesh, India, Indonesia, Pakistan, Botswana, Kenya, Mauritius, South Africa, Swaziland, Tanzania, Uganda, Zimbabwe) Global North (Singapore)	Cross-sectional Qualitative study	*n* = 22 (13 females) sibs; *M*_age_ = n.s.; Age range = 8 – 25 yo	Mental disorders (i.e., intellectual and developmental disabilities)	Three semistructured focus group	Three main themes were revealed: (1) benefits; (2); challenges (3) support needed. On one hand, Sibs experienced challenges because of the management of his/her brother/sister with disabilities, his/her family, and social stigma connected to disability On the other hand, sibs referred a positive/close sibs relationship and a personal growth because of the sibs relationship In addition, other themes of the focus groups regarding the e future planning and support services designed for sibs	High
2	Guidotti et al., 2021	Global North (Italy)	Cross -sectional Mixed study	*n* = 44 (25 females) sibs *M*_age_ = 8.97 (3.96) yo Age range = 6 – 17 yo	Mental disorders (i.e., autism)	SRI (sibling relationship); Drawings about sibling relationship	Results showed that the sibs relationship was characterized by warmth and closeness Drawings analysis revealed conflict in family environment. Sibs reported themselves as experienced negative emotions and the brother/sister with disability was represented as experienced positive emotions	High
3	Rossetti et al., 2020	Global North (USA)	Cross-sectional Qualitative study	*n* = 8 (7 females) sibs; *M*_age_ = n.s Age range = 22–47 yo	Mental disorders (i.e., intellectual and developmental disabilities) & Genetic disease (i.e., Down syndrome)	Dyadic semi-structured interviews developed for the study purposes (sibling relationship)	Sibs reported a comfortable relationship. Nevertheless, the dyad sibs-brother/sister with Down syndrome was characterized by greater reciprocity than the dyad sibs-brother/sister with autism	Medium
4	Travers et al., 2020	Global North (USA)	Cross-sectional Quantitative study	*n* = 155 (122 females) sibs; *M*_age_ = 23.2 (3.8) yo; Age range = 18–30 yo Specifically: *n* = 77 Sibs of brother/sister with autism; *n* = 97 Sibs of brother/sister with intellectual disabilities; *n* = 19 Sibs of brother/sister with ASD and ID	Mental disorders (i.e., autism, intellectual disabilities)	Questions developed for the study purposes (joint activities); Questions extracted from Positive Affect Index (sibling relationship); Questions developed for the study purposes (expectations)	Sibs reported that they spend a lot of time with their brother/sister with disabilities. They also described their sibs relationship as positive The severity of the brother/sister disabilities is negatively associated with the sibs relationship	Medium
5	Avieli et al., 2019	Global North (Israel)	Cross-sectional Qualitative study	*n* = 15 (gender distribution n.s.) sibs; *M*_age_ = n.s Age range = 25 – 62 yo	Neurological disease (Cerebral Palsy)	In-depth semi-structured interviews developed for the study purposes (sibs’ role in family, sibs relationship, family interactions)	Content analysis revealed five patterns of sibs relationships: (a) sibs as the surrogate parent; (b) the estranged sib; (c) the bystander sib; (d) the mediator sib; and (e) the friend sib	High
6	Hemati Alamdarloo et al., 2021	Global South (Iran)	Cross-sectional Quantitative study	*n* = 23 (13 females) sibs of brother/sister with blindness (experimental group); *M*_age_ = 14.13 (2.80) yo Age range = 10 – 18 yo *n* = 15 (9 females) sibs of brother/sister with deafness (experimental group); *M*_age_ = 13.80 (2.79) yo; Age range = 10 – 18 yo *n* = 53 (26 females) sibs of TD individuals (control group); *M*_age_ = 14.54 (2.66) yo; Age range = 10 – 18 yo	Sensory diseases (blindness; deafness)	SRQ (sibling relationship)	Results showed a more conflictual sibs relationship for sibs of people with sensory diseases than sibs of TD individuals	Medium
7	Shivers, 2019a	Global North (USA)	Cross-sectional Quantitative study	*n* = 26 (14 females) sibs (experimental group); *M*_age_ = 14.54 (1.96) yo Age range = 12 – 18 yo *N* = 48 (33 females) sibs of TD individuals (control group) *M*_age_ = 14.27 (2.01) yo Age range = 12 – 18 yo	Mental disorders (i.e., autism)	IRI (sib empathy); MAACL-R (sib relationship); Three wishes (empathy)	Sibs of brother/sister with autism showed greater perspective-taking compared to sibs of TD individuals No difference regarding empathy was found Sibs of brother/sister with autism reported high levels of empathy and positive feelings toward the brother/sister with disabilities Sibs of TD people reported wishes for themselves	Medium
8	Noonan et al., 2018	Global North (Ireland)	Cross-sectional Qualitative study	*n* = 8 (5 females) sibs *M*_age_ = n.s Age range = 18 – 37 yo	Mental disorders (i.e., autism)	In-depth semi-structured interviews developed for the study purposes (sibs relationship, family dynamics, future)	Results showed that sibs of brother/sister with disabilities reported contradictory emotions: on one hand, they reported worry and relationship difficulties; on the other hand, they reported gratitude for personal growth	High
9	Yacoub et al., 2018	Global North (Ireland)	Cross-sectional Qualitative study	*n* = 11 (gender distribution n.s.) sibs *M*_age_ = n.s Age range = n.s	Mental disorders (i.e., autism)	Interview extracted from the Parenting stress Index (impact of the autistic symptomatology)	Results showed that more adaptive behaviors were required to sibs when a autism occurred. Furthermore, sibs reported worry about the future, stress, feelings of anxiety, and guilt	High
10	Tyerman et al., 2019	Global North (UK)	Cross-sectional Qualitative study	*n* = 5 (1 female) sibs *M*_age_ = n.s Age range = 9 – 12 yo	Neurological diseases (acquired brain injury)	Semi-structured interviews developed for the study purposes (sibling relationship)	Sibs reported high levels of distress and anxiety because of the high likelihood of death of the brother/sister The severity of the disabilities affecting the brother/sister led sibs to experience uncertainty about the future and the sibling relationship	High
11	Bhattashali et al., 2018	Global South (India)	Cross-sectional Qualitative study	*n* = 12 (8 females) sibs *M*_age_ = 12.2 (n.s.) yo Age range = 9 – 16 yo	Mental disorders (i.e., developmental disabilities)	Interview developed for the study purposes (sibling relationship, knowledge, and attitudes regarding the disabilities of the brother/sister)	Results showed that sibs spend a lot of time with the brother/sister with disabilities In addition, they reported a positive quality of sibs relationship	High
12	Braconnier et al., 2018	Global North (USA)	Cross-sectional Quantitative study	*n* = 37 (29 female) sibs *M*_age_ = 9.18 (1.90) yo Age range: = 6 – 13 yo	Mental disorders (i.e., autism)	SRQ (sibling relationship)	Sibs perceived the sib’s relationship as low conflictual than parents	High
13	Corsano et al., 2017	Global North (Italy)	Cross-sectional Qualitative study	*n* = 14 (5 females) sibs *M*_age_ = 6.07 (2.46) yo Age range = 12–20 yo	Mental disorders (i.e., autism)	Semi-structured interview developed for the study purposes (sibling relationship and attitude toward brother/sister with disabilities)	Sibs showed ambivalent feelings On the one hand, they reported excessive responsibility, future concerns, stress, embarrassment, relationship difficulties On the other hand, they reported positive feelings increasing over time	High
14	Tomeny et al., 2017a, 2017b	Global North (USA)	Cross-sectional Quantitative study	*n* = 82 (69 females) sibs Specifically: *n* = 45 (37 females) sibs of brother/sister with autism *M*_age_ = 29.42 (11.08) yo Age range = 18–62 yo *n* = 37 (32 females) sibs of individuals with intellectual disabilities *M*_age_ = 36.30 (13.07) yo Age range = 19–61yo	Mental disorders (i.e., autism; intellectual disabilities)	DASS (distress); LSRS (sibling relationship); SWLS (satisfaction with life)	Sibs of brother/sister with autism reported fewer positive attitudes in sibs’ relationships than sibs of individuals with intellectual disabilities	High
15	Cuskelly, 2016	Global North (Australia)	Cross-sectional (i.e., follow up of a longitudinal study; [101;22]) Quantitative study	*n* = 39 (23 females) sibs; *M*_age_ = 28.8 (2.83) yo; Age range = 22–35 yo	Genetic disease (i.e., Down syndrome)	ASRQ (sibling relationship); The Impression Management Scale (influence of social desirability of Sibs responses)	Behavioral problems of the brother/sister with Down syndrome predicted the warmth of the sibs relationship during adulthood Sibs perceived the sibling relationship as warm and no conflictual	High
16	Floyd et al., 2016	Global North (USA)	Cross-sectional & Longitudinal Quantitative study	*Cross-sectional sample* *n* = 106 (69 females) sibs; *M*_age_ = 22.92 (5.81) yo; Age range = 11—38 yo *Longitudinal sample* *n* = 35 (gender distribution n.s.) sibs; *M*_age_ (Wave 1) = 13.49 (3.02) yo; Age range = 10–22 yo *M*_age_ (Wave 2) = 5.91 (3.03 yo; Age range = 22–34 yo	Mental disorders (i.e., intellectual disabilities)	SRQ (sibling relationship)	Sibs of brother/sister with disabilities reported both closeness and conflict in sib relationships Sibs’ age impacted across time. In other words, from adolescence to young adulthood the emotional closeness increased and the conflict reduced	Medium
17	Stock et al., 2016	Global North (UK)	Cross-sectional Qualitative study	*n* = 5 (2 females) sibs; *M*_age_ = 17 (n.s.) yo; Age range = 11–31 yo	Genetic disease (i.e., cleft lip and/or palate)	Open-ended and semi-structured interview developed for the study purposes	Sibs reported a strong sibling relationship In addition, they reported high level of responsibility toward their brother/sister with disabilities. Sibs reported high levels of kindness and nonjudgmental attitude to others Nevertheless, sibs identified rivalry in sibling relationship because of parental attention to their brother/sister with disabilities	High
*Siblings-focused Parentification*								
1	Brolin et al., 2024	Global North (Italy, Netherlands, Slovenia, Sweden, Switzerland, UK)	Cross-sectional Quantitative study	*n* = 467 (gender distribution n.s.) sibs; *M*_age_ = n.s.; Age range = 15–17 yo	Health-related condition (physical disabilities, mental illness, cognitive impairments, addiction, or other health-related conditions)	(MACA-YC18 (amount of caring activities); Kidscreen-10 (Ravens-Sieberer et al., 2014; health-related quality of life); PANOC-YC20 (outcomes of care provision)	Sibs reported positive aspects of caring, such as increased maturity, and negative ones as well, such as mental ill-health, impact on schooling and a lack of support	High
2	eun Lee et al., 2023	Global North (South Korea, Japan, Taiwan)	Cross-sectional Quantitative study	*n* = 576 (415 females) sibs; *M*_age_ = 26.41 (0.51) yo; Age range = 18–48 yo Specifically: *n* = 170 (108 females) Korean sibs; *M*_age_ = n.s.; Age range = 18–48 yo *n* = 308 (240 females) Japanese sibs; *M*_age_ = n. s.; Age range = 18–48 yo *n* = 98 (67 females) Taiwanese sibs; *M*_age_ = n.s.; Age range = 18–48 yo	Mental disorders (i.e., intellectual and developmental disabilities)	ADL (levels of independence of people with disabilities); Scale developed by Horowitz (Horowitz, 1985; caregiving); Scale developed by Perkins and Haley (Perkins & Haley, 2010; difficulties due to the caregiving responsibility); Three items from the Advocacy Scale developed by Taylor and colleagues (Taylor et al., 2017; advocacy); A single item about the number of caregiving supporters (Penrod et al., 1995); Questions about future caregiving competence (Pearlin et al., 1990); Future Planning Scale (future planning); Question about future planning barriers (Burke et al., 2018)	The study compared the sibs’ parentification across three cultures (South Korea, Japan, and Taiwan). The total sample reported low engagement in advocacy activities and low level of perceived caregiving Korean sibs reported more negative perceptions of caregiving than Japanese and Taiwanese sibs Japanese sibs reported no involvement in future planning compared to Korean and Taiwanese sibs Taiwanese sibs reported higher levels of engagement in caregiving than Korean and Japanese sibs	High
3	Niedbalski, 2023	Global North (Poland)	Cross-sectional Qualitative study	*n* = 20 (10 females) sibs *M*_age=_ 25.95 (7.0) yo; Age range = 16–37 yo	Mental Disorders (intellectual disabilities)	Open-ended questions developed for the study purposes about their life experience related to having a brother/sister with disability	Most siblings reported that they were willing to take on the role of caregiver for their brother/sister, although many siblings experienced this decision as an obligation, and they expressed concern. In addition, siblings emphasized the importance of open communication with parents about the management of the sibling's disability and the future	High
4	Chiu, 2022	Global South (China)	Cross-sectional Qualitative study	*n* = 30 (21 females) sibs; *M*_age_ = n.s Age range = 20–66 yo	Mental disorders (i.e., intellectual and developmental disabilities)	In-depth interview developed for the study purposes (roles served by sibs)	Based on the thematic analysis, results revealed a sibling relationship characterized by love and hate simultaneously Sibs reported anxious traits because of the lack of communication about caregiving tasks with their parents. Furthermore, sibs reported as a mandatory task the caregiving toward their brother/sister with disabilities Different sibs’ roles were detected: minimal caregiving role, anticipating caregiver role, and primary/joint caregiver role	High
5	Kale & Siğirtmaç, 2021	Global North (Turkey)	Cross-sectional Qualitative study	*n* = 6 (3 females) Sibs of brother/sister with Down syndrome, congenital visual disability, and orthopedic inadequacy; *M*_age_ = n.s Age range = n.s	Genetic disease (i.e., Down Syndrome; Congenital visual disability) & Motor disorders (Orthopedic inadequacy)	Interview developed for the study purposes (roles served by sibs)	Results highlighted that the sibs served as caregiver regardless the sibs gender Caregiving increased according to age affecting the sibs social interaction with peers	Medium
6	Beffel & Nuttall, 2020	Global North (USA)	Cross-sectional Quantitative study	*n* = 108 (75 females) sibs; *M*_age_ = 20.37 (1.55) yo; Age range = 18–24 yo	Mental disorders (i.e., autism)	PI (parentification); Benefit Finding (perceived benefits of parentification); PS (prosocial and helping behaviors); BAPQ (autistic features)	Results showed that sibs-focused parentification negatively predicted prosocial behavior when low benefits of parentification were perceived	High
7	eun Lee et al., 2020	Global North (USA)	Cross-sectional Mixed study	*n* = 332 (286 females) sibs; *M*_age_ = 35.94 (13.57) yo; Age range = 18–75 yo; Specifically: *n* = 152 (130 females) non-caregivers sibs; *M*_age_ = 33.06 (11.78) yo *n* = 94 (77 females) single caregivers sibs; *M*_age_ = 32.27 (13.44) yo *n* = 86 (81 females) compound caregivers sibs; *M*_age_ = 45.38 (12.43) yo	Mental disorders (i.e., intellectual and developmental disabilities)	Open-ended questions developed for the study purposes (sibling relationship, roles served by the sibs, future, and support); PAI (sibling relationship)	Single and compound caregivers reported greater level of advocacy than non-caregiver sibs Furthermore, they reported future planning compared to non-caregivers Sibs	High
8	Redquest et al., 2020	Global North (Canada)	Cross-sectional Quantitative study	*n* = 260 (gender distribution n.s.) sibs; *M*_age_ = n.s Age range = 20–29 yo (*n* = 119 Sibs); Age range = 30–49 yo (*n* = 94 Sibs); Age range = 50 or older (*n* = 47 Sibs)	Mental disorders (i.e., intellectual and developmental disabilities)	Questionnaire developed for the study purposes (current and future support role, support challenges, desired resources, resources access)	Sibs reported that they have had a marginal role in supporting their own brother/sister with disabilities. Nevertheless, the majority of them reported that they will plan to serve a caregiver role in the future	High
9	Brady et al., 2019	Global North (USA)	Cross-sectional Qualitative study	*n* = 10 (7 females) sibs; *M*_age_ = n.s.; Age range = 20–68 yo	Mental disorders (i.e., intellectual and developmental disabilities)	ADLs (level of independence of people with disabilities); Interview (Sibs’ role in caregiving and guardianship)	The study allowed to theorize the Sibling Reciprocal Effect, that is the phenomenon of siblings to recognize the applicability of complementary forms of guardianship for other adults with mental disorders. Nevertheless, authors deferred to full guardianship as the preferred mechanism for decision making	High
10	eun Lee et al., 2019a, 2019b	Global North (USA)	Cross-sectional Quantitative study	*n* = 429 (378 females) sibs (total sample); *M*_age_ = 37.27 (13.94) yo; Age range = 18–75 yo *n* = 218 (199 females) sibs (restricted sample); *M*_age_ = 38.87 (14.67) yo; Age range = 18–75 yo	Mental disorders (i.e., intellectual and developmental disabilities)	Scales of Independent Behavior‐Revised (maladaptive behaviors); Activities of Daily Living scale (levels of independence of people with disabilities); Positive Affect Index (sibling relationship); Open-ended question developed for the study purposes (parent caregiving ability); Three dependent variables (Sibs caregiving status, time spent in caregiving, nature of caregiving)	Sibs caregiving was associated to the quality of sibling relationships, sibling advocacy, future planning. Furthermore, a good caregiving served by the sibs was correlated with low maladaptive behaviors in brother/sister with mental disorders	High
11	eun Lee et al., 2019a, 2019b	Global North (USA)	Cross-sectional Quantitative study	*n* = 141 (126 females) sibs; *M*_age_ = 55.87 (8.48) yo; Age range = 33–78 yo Specifically: *n* = 53 Sibs of brother/sister with intellectual disabilities; *n* = 42 Sibs of brother/sister with developmental delay; *n* = 33 Sibs of brother/sister with motor disability; *n* = 30 Sibs of brother/sister with Down syndrome; *n* = 26 Sibs of brother/sister with autism	Mental disorders (i.e., intellectual disabilities; developmental delay; autism) & Motor disabilities & Genetic diseases (Down Syndrome)	ADL (functional abilities of the person with disabilities); SIB-R (maladaptive behaviors); Scale developed by Horowitz (Horowitz, 1985; caregiving); Scale developed by Perkins and Haley (Perkins & Haley, 2010; difficulties due to the caregiving responsibility); Scale developed by Taylor and colleagues (Taylor et al., 2017; advocacy); Questions developed for the study purposes (caregiving)	Maladaptive behaviors in brother/sister with disabilities was associated to high requests for assistance Sibs of older brothers/sisters with disabilities showed engaged in caregiving, advocacy, and future planning	High
12	eun Lee et al., 2018	Global North (USA)	Cross-sectional Qualitative study	*n* = 70 (53 females) sibs; *M*_age_ = 43.13 (16.78) yo; Age range = 18–75 yo	Mental disorders (i.e., intellectual disabilities)	Adult Sibling Survey (sibling relationship; future planning; caregiving); Open-ended question developed for the study purposes (sib’s roles)	Results suggested that albeit the sibs did not identify themselves with the role of caregiver, 79% of them performed pivotal caregiving tasks for their brother/sister with disabilities	High
13	Lashewicz, 2018	Global North (Canada)	Cross-sectional Qualitative study	*n* = 5 (4 females) sibs; *M*_age_ = n.s Age range = 22–38 yo	Mental disorders (i.e., developmental disabilities)	In-depth focus group interview (supporting roles played by Sibs)	Thematic analysis reported that sibs roles were (1) companion and protector; (2) follower; (3) caregiver; (4) family protector	High
14	Nuttall et al., 2018	Global North (USA)	Cross-sectional Quantitative study	*n* = 108 (75 females) sibs; *M*_age_ = 20.37 (1.55) yo; Age range = 18–25 yo	Mental disorders (i.e., autism)	PI (parentification); Autism Benefit Finding Scale (perceived benefits from parentification); Intention for Caregiving Involvement in the Future (future intention for caregiving involvement)	Results showed that sibs who perceived more benefits having and growing with a brother/sister with autism were inclined to future planning	High
15	Rossetti et al., 2018	Global North (USA)	Cross-sectional Mixed study	*n* = 171 (140 females) sibs; *M*_age_ = n.s Age range = 18–72 yo	Mental disorders (i.e., intellectual and developmental disabilities	Open-ended questions developed for the study purposes (sib’s roles)	Findings identified 7 sibs’ roles, that is caregiver, friend, advocate, legal representative, sibling, leisure planner, informal service coordinator	High
16	Tomeny et al., 2017a, 2017b	Global North (USA)	Cross-sectional Quantitative study	*n* = 41 (33 females) sibs; *M*_age_ = 25.83 (5.36) yo; Age range = 18 – 37 yo	Mental disorders (i.e., autism)	PI (parentification); LSRS (sibling relationship); DASS-21(distress)	Findings showed that sib-focused parentification was positively correlated with stress as well as to positive sibling relationship	High
17	Tomeny et al., 2016a, 2016b	Global North (USA)	Cross-sectional Quantitative study	*n* = 60 (51 females) sibs; *M*_age_ = 29.65 (13.17) yo; Age range = 18–68 yo	Mental disorders (i.e., autism)	PI (parentification); ISEL (perceived availability of social support); DASS-21 (distress); LSRS (sibling relationship)	Social support perceived by sibs moderated the interplay between sibs-focused parentification and the sibs relationship: the lower sib-focused parentification, the lower support, the less positive sibs relationships	High
*Emotional and Behavioral Adjustment*								
1	Hayden et al., 2023	Global North (UK)	Cross-sectional Quantitative study	*n* = 852 (720 females) sibs; *M*_age_ = 34.75 (12.76) yo; Age range = 18–76 yo	Mental disorders (i.e., intellectual and developmental disabilities)	K6 (distress); SWEMWBS (wellbeing); Questions about quality of life and health (Information Centre for Health and Social Care, GfK NOP, 2011); Questions developed for the study purpose (caregiving); Question about subjective poverty (Australian Institute of Family Studies, 2011); Question about financial management (MCS, 2017); W-ADL (level of independence in daily living of the brother/sister with disability)	The results show that sibs with brother/sister with higher levels of independence experienced less distress, high levels of wellbeing, and high quality of life In addition, the study found a moderating effect of the individuals’ status socio-economic on the interplay between sibs’ career status and their levels of distress and wellbeing	High
2	Siman-Tov & Sharabi, 2023	Global North (Israel)	Cross-sectional Quantitative study	*n* = 99 (63 females) sibs; Specifically: *n* = 59 Sibs of people with intellectual disability; *n* = 40 Sibs of people with autism; *M*_age_ = 29.73 (8.38) yo; Age range = 18 – 62 yo	Mental Disorders (intellectual disability or autism)	Self-efficacy (Chen et al., 2001; self-efficacy); Sense of Coherence Scale (Antonovsky, 1993; sense of coherence); Loneliness Scale (Davidson et al., 2012; emotional and social loneliness); Mental Health Inventory (Veit & Ware, 1983; mental wellbeing and distress)	Regarding gender differences, sisters reported significantly higher levels of involvement, self-efficacy and wellbeing than brothers; brothers reported significantly higher levels of loneliness than sisters. In addition, sisters of people with intellectual disability reported more involvement than brothers. Regarding disability differences, siblings of people with autism reported less distress than siblings of people with intellectual disabilities	Medium
3	Hanvey et al., 2022	Global North (UK, Ireland, Germany)	Cross-sectional Qualitative study	*n* = 16 (14 females) sibs; *M*_age_ = 33.25 (14.31) yo; Age range = 20–68 yo	Mental disorders i.e., autism, learning disorder) & Neurological disease (injury, epilepsy)	Semi-structured interview developed for the study purposes	The thematic analysis identified four themes: (1) feelings of invisibility during social interactions, (2) psychological difficulties; (3) feelings of guilt and self-blame; (4) social support Specifically, sibs reported a lack of attention from family and negative feelings, in terms of anxiety and worry, guilt	High
4	Milevsky & Singer, 2022	Global North (North America)	Cross-sectional Qualitative study	*n* = 20 (17 females) sibs; *M*_age=_ 31.35 (10.30) yo; Age range = 20–57 yo	Mental disorders (i.e., developmental disabilities) & Genetic disease (i.e., Down syndrome)	Semi-structured interview developed for the study purposes	Thematic analysis revealed as relevant themes the stress and the quality of life; the influence on romantic relationships; planning for the future Sibs reported high levels of general difficulties and anxiety, feelings of social awkwardness of concern about the future. However, sibs also referred high level of empathy, kindness, acceptance of others	High
5	Orm et al., 2022	Global North (Norway)	Cross-sectional Quantitative study	*n* = 47 (18 females) sibs of brother/sister with mental disorders; (experimental group); *M*_age_ = 11 (2.3) yo; Age range = 8–16 yo *n* = 42 (19 females) sibs of brother/sister with motor disabilities (experimental group); *M*_age_ = 11.5 (2.0) yo; Age range = 8–16 yo *n* = 44 (27 females) sibs of TD individuals (control group); *M*_age_ = 11.4 (2.5) yo; Age range = 8–16 yo	Mental disorders (i.e., autism) & Motor disabilities	SDQ (emotional and behavioral adjustment); NAS (negative adjustment to brother/sister disability); PCCS-C (openness and emotional support in parent–child communication)	Both experimental groups showed higher levels of prosocial behavior than the control group. Sibs’ externalizing/internalizing difficulties and mother–child communication were associated: in other words, the more open mother–child communication is the more sibs’ prosocial behavior	High
6	Le Boudec et al., 2021	Global North (Switzerland)	Cross-sectional Quantitative study	*n* = 1.567 (717 females) sibs (experimental group); Females *M*_age_ = 17.8 (.05) yo; Males *M*_age_ = 17.6 (.5) yo Age range = 16–25 yo *n* = 145 (64 females) sibs of TD individuals (control group); Females *M*_age_ = 17.5 (.1) yo; Males *M*_age_ = 17.6 (.2) yo Age range = 16–25 yo	Not reported	WHO-5 (wellbeing); Perceived Stress Scale (stress); SMASH-02 (perceived health status); SCOFF questionnaire (eating disorder); Questionnaire developed for the study purposes (externalizing behaviors)	Compared to the control group, female sibs of brother/sister with disability reported more somatic problems, more smoking dependence than their counterpart Males sibs of brother/sister with disabilities reported more aggressive behaviors than female ones No significant differences on emotional wellbeing, stress level, school performances, and substance use (except tobacco) between groups were found	Medium
7	Yaldız et al., 2021	Global South (Turkey)	Cross-sectional Quantitative study	*n* = 72 (47 females) sibs (experimental group); *M*_age_ = 18.65 (1.44) yo; Age range = 16–21 yo *n* = 109 (94 females) sibs of TD individuals (control group); *M*_age_ = 18.92 (1.55) yo; Age range = 16–21 yo	Mental disorders (i.e., developmental disabilities)	YSQ-SF3 (maladaptive behaviors); GSJS (level of system justification); Questionnaire developed for the study purposes (negative emotions)	No significant differences regarding negative emotions between the two groups were found Sibs of individuals with mental disorders showed more interest in other’s desires and needs and the level of system justification was higher than the control group	Medium
8	Shojaee et al., 2020	Global South (Iran)	Cross-sectional Quantitative study	*n* = 49 (24 females) sibs of brother/sister with autism (experimental group 1); *M*_age_ = 14.16 (2.21) yo; Age range = 10–18 yo *n* = 42 (27 females) sibs of brother/sister with intellectual disability (experimental group 2); *M*_age_ = 14.76 (2.58) yo; Age range = 10–18 yo *n* = 50 (33 females) sibs of TD individuals (control group) *M*_age_ = 14.40 (2.33) yo; Age range = 10–18 yo	Mental disorders (i.e., autism, intellectual disability)	SDQ (emotional and behavioral adjustment)	Sibs of brother/sister with mental disorders reported higher score in maladjustment compared to sibs of TD individuals Sibs of brother/sister with autism reported high emotional problems than sibs of TD people Sibs of brother/sister with mental disorders showed more hyperactive behaviors than sibs of TD people Sibs of people with mental disorders showed more difficulties in peer relationship than sibs of TD individuals No significant difference was found between the three groups of sibs in terms of conduct problems	Medium
9	Shivers, 2019b	Global North (USA)	Cross-sectional Quantitative study	*n* = 1.021 (805 females) sibs; *M*_age_ = 36.85 (13.71) yo; Age range = n.s	Mental disorders (i.e., intellectual and developmental disabilities)	PAI (sibling relationship); ADL (functional abilities of the brother/sister with disability); CES-D (five questions- depressive symptoms); Questions developed for the study purposes (perceived health status); Questions developed for the study purposes (perceived sense of guilt)	Over 50% of siblings reported the experience of having a sibs with disabilities as characterized by guilt Guilt was related to a weak sibling relationship, depressive symptoms, and lower levels of wellbeing In addition, guilt was associated with the severity of brother/sisters emotional and behavioral problems	Medium
10	Perenc & Pęczkowski, 2018	Global North (Poland)	Cross-sectional Quantitative study	*n* = 128 (72 females) sibs (experimental group); *M*_age_ = 15.77 (1.61) yo; Age range = 13–19 yo *n* = 164 (90 females) sibs of TD individuals (control group); *M*_age_ = 15.82 (1.58) yo; Age range = 13–19 yo	Motor disabilities	IRI (empathy)	Sibs of people with motor disabilities showed higher levels of cognitive and emotional empathy than those of TD individuals Female sibs reached higher levels of empathy compared to their counterparts	High
11	Shivers & Kozimor, 2017	Global North (USA)	Cross-sectional Quantitative study	*n* = 9 (5 females) sibs of brother/sister with mental disorder and co-occurring (experimental group); *M*_age_ = 15.22 (1.79) yo; Age range = 12–18 yo *n* = 40 (20 females) Sibs of brother/sister with mental disorders (control group); *M*_age_ = 14.25 (1.94) yo; Age range = 12–18 yo	Mental disorders (i.e., intellectual and developmental disabilities with and without co-occurring)	MAACL-R (emotions toward brother/sister with disability)	Sibs of brothers/sisters with mental disorders and co-occurring reported higher levels of hostility, anxiety, and dysphoria than sibs with only mental disorders No difference in positive affects between groups were found	Medium
12	O’Neill & Murray, 2016	Global North (UK)	Cross-sectional Quantitative study	*n* = 132 (98 females) sibs (experimental group); Age range = 19 – 71 yo Specifically: *n* = 59 Sibs of people with Down Syndrome *M*_age_ = 33.22 (9.01) yo; *n* = 31 Sibs of people with autism *M*_age_ = 32.19 (11.52) yo; *n* = 26 HS of people with Prader-Willi Syndrome *M*_age_ = 30.65 (9.70) yo; *n* = 16 HS of people with disability of unknown etiology *M*_age_ = 42.50 (14.59) yo *n* = 132 (98 females) Sibs of TD (control group); *M*_age_ = 37.03 (12.34) yo Age range = 19 – 71 yo	Mental disorders (i.e., autism) & Genetic disease (Down Syndrome; Prader-Willi Syndrome) Disability of unknown etiology	HADS (anxiety)	The results showed that sibs of people with autism and Prader-Willi syndrome reported higher level of anxiety than sibs of TD individuals Sibs of people with autism and disability with unknown etiology reported higher levels of depression than the control group No difference in anxiety and depression between sibs of people with Down syndrome and the control group were found. Female sibs of brother/sister with Down syndrome reported higher levels of anxiety than their counterpart. Sibs’ age was negatively correlated with depressive symptoms	Medium
13	Tomeny et al., 2016a, 2016b	Global North (USA)	Cross-sectional Quantitative study	*n* = 56 (all females) sibs; *M*_age_ = 13.21(1.93) yo; Age range = 11–17 yo	Mental disorders (i.e., autism)	SDQ (emotional and behavioral adjustment)	The severity of autistic symptomatology was correlated with the sibs’ maladjustment	High
14	Tsai et al., 2016	Global North (Taiwan, UK)	Cross-sectional Quantitative study	*Taiwan sample*: *n* = 80 (47 females) sibs; *M*_age_ = 12.7 (2.8) yo; Age range = 7 -18 yo *UK sample*: *n* = 75 (47 females) sibs; *M*_age_ = 12.7 (2.4) yo; Age range = 8 -17 yo	Mental disorders (i.e., autism)	CASE (impact of stressful life events); SWLS (life satisfaction); CASSS (social support); Kidcope (coping strategies); SDQ (emotional and behavioral adjustment)	The emotional and behavioral adjustment of Taiwanese sibs was better than UK ones The good impact of life experiences, good coping strategies, and high social support were associated with sibs emotional and behavioral adjustment	Medium
*Well-Being*								
1	Lahaije et al., 2023	Global North (Netherlands)	Cross-sectional Quantitative study	*n* = 18 (12 females) sibs; Females *M*_age_ = 16.3 (2.8) yo; Males *M*_age_ = 15.2 (2.8) yo Age range = 12 – 20 yo	Mental Disorders (intellectual disabilities)	Beach Center FQOL Scale (wellbeing; Hoffman et al., 2006)	The study compared levels of well-being reported by parents with those reported by siblings. The results indicate that siblings report significantly higher scores than parents on the emotional well-being, material well-being, and disability-related support	High
2	Correia & Seabra-Santos, 2022	Global North (Portugal)	Cross-sectional Qualitative study	*n* = 6 Sibs (5 females) sibs; *M*_age_ = 34.67 (19.38) yo; Age range = 17–56 yo	Mental disorders (i.e., autism) & Genetic disease (e.g., Down syndrome, X fragile)	In-depth interviews	Sibs referred that having a brother/sister with disabilities promoted the personal growth, in terms of maturity, tolerance, more understanding, and patience In addition, sibs reported limitations imposed by the disabilities of their brother/sister. Nevertheless, they revealed a sense of responsibility and protection toward their family members	High
3	Koukouriki & Soulis, 2020	Global North (Greece)	Cross-sectional Quantitative study	*n* = 118 (59 females) sibs (experimental group); *M*_age_ = n.s.; Age range = 9–13 yo *n* = 115 (59 females) sibs of TD individuals (control group); *M*_age_ = n.s.; Age range = 9–13 yo	Mental disorders (i.e., autism)	Kidscreen‐27 (Ravens-Siebrer et al., 2007; Quality of life); STAIC, A‐Trait (anxiety); GHQ-28 (general health); MSPSS (social support)	Sibs of brother/sister with mental disorders showed lower levels of wellbeing and higher levels of anxiety than sibs of TD individuals Sibs’ wellbeing of brother/sister with disabilities was associated with family support Sibs’ anxiety was associated with parental anxiety	High
4	Hallion et al., 2018	Global North (Australia)	Cross-sectional Quantitative study	*n* = 65 (44 females) sibs (experimental group); *M*_age_ = 22.66 (7.97) yo; Age range = 17–61 yo *n* = 79 (40 females) sibs of TD individuals (control group); *M*_age_ = 19 (1.66) yo; Age range = 17–26 yo	Chronic illness and disability not specified	PWB (psychological well-being); DASS-21 (distress)	No significant differences in wellbeing and distress levels between sibs of brother/sister with disabilities and those of TD people were found	High
*Hybrid Studies*								
1	Kulisch et al., 2024	Global North (Germany)	Cross-sectional Quantitative study	*n* = 81 (54 females) sibs; *M*_age=_ 10.8 (2.2) yo; Age range = 6–16 yo	Not Specified	SVF-KJ (coping strategies); KIDSCREEN-10 (Ravens-Sieberer et al., 2010; quality of life)	Cluster analysis identified two behavioral patterns: high coping (37%) and low coping (63%). In both cases, patterns demonstrated no significant difference in terms of quality of life Coping strategies such as minimization, situation control, positive self-instructions, and the need for social support were positively associated to quality of life	High
2	Levante et al., 2023a, 2023b, 2023c	Global North (Italy)	Cross-sectional Quantitative study	*n* = 605 (570 females) sibs; *M*_age=_ 22.49 (2.91) yo; Age range = 19–26 yo	Not Specified Genetic diseases & Motor disabilities & Mental disorders (with and without co-occurring) & Sensory disease	PI (parentification); DASS-21 (distress); MSPSS (perceived social support); Questions developed for the study purpose (sibling relationship); Questions developed for the study purposes (sibs-parents relationship)	Results revealed that high sibs’ distress and negative quality of sibs-parents relationship negatively affected the interplay between the sibs’ parentification and the sibs relationship Perceived benefits of parentification and perceived social support decreased the sibs’ distress levels	High
3	Zaidman-Zait et al., 2020	Global North (Israel)	Cross-sectional Quantitative study	*n* = 28 (14 females) sibs (experimental group); *M*_age_ = 10.40 (1.3) yo; Age range = 8–13 yo *n* = 31 (15 females) sibs of TD individuals (control group); *M*_age_ = 9.90 (1.3) yo; Age range = 8–13 yo;	Mental disorders (i.e., intellectual disabilities)	SDQ (emotional and behavioral adjustment); SRQ (sibling relationship); Drawings (sibling relationship)	Sibs of brother/sister with mental disorders showed higher levels of empathy, lower levels of conflict, and rivalry compared to sibs of TD individuals Sibs relationship was associated with children's adjustment Drawings revealed that sibs of brother/sister with disabilities showed more support and investment in the sibling relationship	High
4	Cebula et al., 2019	Global North (UK; USA; Australia; Canada)	Cross-sectional Quantitative study	*n* = 31 (24 females) sibs; *M*_age_ = 11.69 (3.16) yo; Age range = 5–17 yo	Genetic disease (i.e., William syndrome)	SDQ (emotional and behavioral adjustment); SCAS (anxiety); SRQ (sibling relationship); SCSS-SV (perceptions of social support)	Sibs showed emotional and behavioral adjustment similar to the general population Conflict in the sibling relationship was associated with sibs behavioral difficulties; warmth in the sibling relationship was negatively associated with the age of the brother/sister with disability, and positively associated with their prosocial behavior	High
5	Jones et al., 2019	Global North (USA)	Cross-sectional Quantitative study	*n* = 52 (16 females) sibs; *M*_age_ = 8.34 (n.s.) Age range = 3.5–18 yo	Mental disorders (i.e., autism)	Children’s Depression Inventory-2nd Edition (depressive symptoms); Autism Knowledge Questionnaire (knowledge about ASD); Questions developed for the study purposes (perceived support); Children’s Coping Strategies Checklist (coping strategies); Sibling relationship questionnaire for siblings (sibling relationship)	Behavioral problems of brother/sister with mental disorders were associated with sibs maladjustment and negative quality of sibling relationship The more positive perception of the sib relationship was associated with high levels of sibs coping strategies and support	High
6	Shivers et al., 2019	Global North (USA)	Cross-sectional Quantitative study	*n* = 215 (115 females) sibs; *M*_age_ = 14.94 (1.75) yo; Age range = n.s Specifically: *n* = 116 (64 females) sibs of brother/sister with autism; *M*_age_ = 15 (1.76) yo *n* = 99 (51 females) sibs of brother/sister with Down syndrome; *M*_age_ = 14.87 (1.74) yo	Mental disorders (i.e., autism) & Genetic diseases (i.e., Down syndrome)	PSS-10 (perceived stress); Questions developed for the study purposes (stress); SRI (sibling relationship); DBC-P24 (behavioral problems in brother/sister with disabilities); MSPSS (Perceived Social Support)	No significant differences were found between groups, in terms of perceived support Sibs of brother/sister with autism reported higher levels of stress sibs of brother/sister with Down syndrome	High
*Psychological impact of brother or sister’s disability on the functioning of their sibling during COVID-19 outbreak*								
1	Redquest et al., 2021	Global North (USA)	Cross-sectional Mixed study	*n* = 91 (86 females) sibs; *M*_age_ = n.s Age range = 20–29 yo (*n* = 26); Age range = 30–39 yo (*n* = 30); Age range = 40–49 yo (*n* = 13); Age range = 50–59 yo (*n* = 15); Age range = 60–69 yo (*n* = 7)	Mental disorders (i.e., intellectual and developmental disabilities)	Questionnaire developed for the study purposes (sibs experiences during COVID-19); Open question developed for the study purposes (helpful resources during COVID-19)	Sibs of brother/sister with disabilities reported that they supported their brother/sister during the pandemic In addition, they reported that they experienced concerns toward their brother/sisters with disabilities because of the disruption of their routines. Furthermore, they experienced concerns that, at times, their brother/sisters with disabilities were unable to accept social distancing Finally, the study showed the benefits of sib’s engagement in self-care activities	Medium
2	Dorsman et al., 2023	Global North (Netherlands)	Cross-sectional Mixed study	*n* = 58 (46 females) sibs; *M*_age_ = 50.1 (12.0) yo; Age range = n.s	Mental disorders (profound intellectual disability)	Questionnaire and open-ended questions developed for the study purposes about sibling roles	Sibs reported taking on multiple roles. The sibling and legal roles were the most common. Although most participants were satisfied with their role, they also reported that the responsibilities made them feel less like siblings. Furthermore, the results indicated that the reduction in contact due to COVID-19 negatively impacted on sibling relationship	High

**Note regarding the measures’ names:** Activities of daily living (ADLs; Lawton et al., 1982); Activities of Daily Living scale (ADL; Seltzer & Li, 1996); Adult Sibling Relationship Questionnaire (ASRQ; Stocker et al., 1997); Advocacy Scale(Taylor et al., 2017); Autism Benefit Finding Scale (Ekas et al., 2015); Autism Knowledge Questionnaire (Kryzak et al., 2015); Autism Parenting Stress Index (Silva & Schalock, 2012); Beach Center FQOL Scale (Hoffman et al., 2006). Benefit Finding (Carver & Antoni, 2004); Broad Autism Phenotype Questionnaire (BAPQ; Hurley et al., 2007); Center for Epidemiological Studies Depression scale (CES-D; Radloff, 1977); Child and Adolescent Social Support Scale (CASSS; Malecki et al., 2000); Child and Adolescent Survey of Experiences (CASE; Allen & Rapee, 2012); Children’s Coping Strategies Checklist (Ayers et al., 1996); Children’s Depression Inventory—2nd Edition (Kovacs, 2011); Depression, Anxiety, and Stress Scale (DASS-21; Lovibond & Lovibond, 1995); Future Planning Scale (Heller & Caldwell, 2006); General Health Questionnaire (GHQ‐28; Garyfallos et al., 1991); General System Justification Scale (GSJS; (Kay et al., 2003); German Coping Questionnaire for Children and Adolescents (SVF-KJ; Hampel & Petermann, 2016); Hospital Anxiety and Depression Scale (HADS; Zigmond & Snaith, 1983); Impression Management Scale (Paulhus, 1991); Intention for Caregiving Involvement in the Future (Skotko et al., 2011); Interpersonal Reactivity Index (IRI; Davis, 1980); Kessler 6 (K6; Kessler et al., 2003); Kidcope measure (Spirito et al., 1991); Lifespan Sibling Relationship Scale (LSRS; Riggio, 2000); Multidimensional Assessment of Caring Activities (MACA-YC18; Joseph et al., 2009); Multidimensional Scale of Perceived Social Support (MSPSS; Zimet et al., 1988); Multiple Affect Adjective Checklist-Revised (MAACL-R; Zuckerman et al., 1985); Negative Adjustment Scale (NAS; Lobato & Kao, 2002); Parent–Child Communication Scale—Child report (PCCS-C; Conduct Problems Prevention Research Group, 1994); Parentification Inventory (PI; Hooper et al., 2011a, 2011b); Perceived Stress Scale (PSS; Cohen & Williamson, 1988); Positive Affect Index of relationship quality (PAI; Bengston & Black, 1973); Positive and Negative Outcomes of Caring (PANOC-YC20; Joseph et al., 2009); Prosocialness Scale (PS; Caprara et al., 2005); Psychological Well-Being scale (PWB; Ryff, 1989); Questionnaire on resources and stress-short form (QRS-F; Friedrich et al., 1983); Satisfaction With Life Scale (SWLS; Diener et al., 1985); Scale of Independent Behavior-Revised (SIB-R; (Bruininks et al., 1996); Short Form of Developmental Behavior Checklist (DBC-P24; Taffe et al., 2007); Sibling Inventory of Behavior (SIB; Hetherington, 1999; Schaeffer & Edgerton, 1981); Sibling Relationship Inventory (SRI; Lecce et al., 2005); Sibling Relationship Questionnaire (SRQ; Buhrmester & Furman, 1990); Spence Children’s Anxiety Scale (SCAS; Spence, 1998); State‐Trait Anxiety Inventory for Children (STAIC, A‐Trait; Spielberger et al., 1973); Strengths and Difficulties Questionnaire (SDQ; Goodman, 2003); Survey of Children’s Social Support: short version (SCSS-SV; Dubow et al., 1997); Swiss multicenter adolescent survey on health 2002 (SMASH 2002; Narring et al., 2004); Waisman Activities of Daily Living Scale (W-ADL; Maenner et al., 2013); Warwick Edinburgh Well-Being Scale (SWEMWBS; Stewart-Brown et al., 2009); WHO-5 well-being index (WHO-5; Topp et al., 2015); Young Schema Questionnaire-Short Form (YSQ-SF3; Young et al., 2003)

**Note regarding other acronyms reported in Table**: Sibs: Siblings of persons with disabilities; TD: typically developing individuals

**Note regarding the methodological quality appraisal of the reviewed studies:** Low: the study reached 1 or 2 “yes” answers; Medium: the study reached 3 “yes”; High: the study reached 4 or 5 “yes” answers

Firstly, the global geographic area (Global North *vs*. Global South) and the Country where each study was conducted were reported. Further methodological details on the study design were extrapolated, in terms of cross-sectional *vs*. longitudinal design, quantitative, qualitative, or mixed data. Additionally, the characteristics of the participants involved in each reviewed study were tabulated: i.e., the total sample size, gender distribution, the type of disability and/or chronic illness of the sibling’s brother or sister, mean age, standard deviation, and age range of the siblings. The types of disabilities and/or chronic illnesses analyzed in the reviewed studies and the measures administered were reported. Finally, the main findings of each reviewed study were resumed and the methodological quality appraisal was reported.

### Methodological Characteristics of the Reviewed Studies

Sixty studies were included in this systematic review. Most of them (*n* = 54) were carried out in Global North countries, five studies involved participants in Global South countries, while only one collected data both in Global North and South countries. Except one (Floyd et al., 2016), all studies (*n* = 59) collected data cross-sectionally. Thirty-seven of them were quantitative, 18 were qualitative, and 4 were mixed studies.

As for the recruited sample, a total of 10,146 participants were involved. The sample size ranged from 9 to 1,567 individuals in quantitative studies, from 5 to 30 participants in qualitative ones, and from 44 to 332 individuals in mixed studies. Eleven studies (Hallion et al., 2018; Hemati Alamdarloo et al., 2021; Koukouriki & Soulis, 2020; Le Boudec et al., 2021; O’Neill & Murray, 2016; Orm et al., 2021; Perenc & Pęczkowski, 2018; Shivers, 2019a; Shojaee et al., 2020; Yaldız et al., 2021; Zaidman-Zait et al., 2020) compared the experimental group including siblings of persons with disabilities to a control group made up of siblings of typically developing individuals (henceforth TD individuals). Forty-nine studies involved experimental group only. As for the gender of the recruited siblings of persons with disabilities, in most of the reviewed studies (*n* = 40), female siblings are more than male counterparts. In eight studies, the sample was gender-balanced, whereas four studies did not provide any detail on the participants’ gender.

The ages of the siblings involved in the reviewed studies ranged from 3 to 78 years of age. Unfortunately, this wide age range does not allow to conduct a sub-analysis (e.g., under and above 18 years) of each psychological construct. Nevertheless, this issue has been discussed in each psychological construct-related section. Except for the studies (*n* = 30) recruiting adult siblings (i.e., ≥ 18 years old) and for four studies that did not provide any information about the participants’ age, it was not possible to cluster participants by developmental stage.

For the specific purposes of the current systematic review, the types of disabilities mentioned in the reviewed studies were grouped in high-order clusters (labels of the cluster were assigned according to Peterson & Keeley, 2015). More specifically, 5 types of clusters were identified: (1) mental disorders (e.g., autism spectrum disorder, intellectual disabilities), (2) motor disabilities (e.g., mobility disabilities), (3) sensory disabilities (e.g., visual impairment, deafness), (4) genetic diseases (e.g., Down syndrome, CHARGE syndrome, 22q11.2 deletion syndrome, Williams-Beuren syndrome), and (5) neurological diseases (e.g., brain injury, epilepsy). As can see in Table 3, the specific type of disability investigated in each empirical study was mentioned in brackets. Resuming, mental disorders were the most investigated condition in the empirical literature (*n* = 39).

Standardized questionnaires were the most used data collection strategy in quantitative studies, while semi-structured interviews were mainly used in qualitative studies, together with open-ended question(s) designed to achieve specific objectives. The main strategies used to identify each psychological construct will be detailed in the following subsections.

### Methodological Quality Appraisal of Included Studies

Results revealed that most of the papers reached a high (*n* = 45) appraisal of their methodological quality, whereas fifteen out of 60 studies reported a medium appraisal quality. Considering the 3-category of studies (i.e., qualitative, quantitative, and mixed) that have been reviewed, the following paragraphs resumed the main results.

*Quantitative studies.* Item 1 of the MMAT assesses whether the sampling strategy is appropriate for addressing the research question(s): All quantitative reviewed studies (*n* = 37) met this criterion. Item 2 evaluates whether the study sample is representative of the target population: 9 out of 37 studies met the criterion. Item 3 examines the appropriateness of the measurement(s) administered to address the research question(s): 33 out of 37 studies used validated and suitable measures. Item 4 assesses the risk of non-response bias: 23 out of 37 studies met this criterion by either maintaining a low non-response rate or using statistical adjustments like the imputation method. Lastly, Item 5 evaluates the appropriateness of the statistical analysis plan: All studies describe in detail statistical analyses computed according to the study design and research question(s). Overall, the quality appraisal of the quantitative studies is medium for 12 studies and high for 25.

*Qualitative studies.* Item 1 evaluates the appropriateness of a qualitative approach in addressing the research question(s): All studies (*n* = 18) met this criterion. Item 2 assesses the adequacy of the data collection method: 14 out of 18 studies met the criterion. Item 3 evaluates whether the data collection methods are aligned with the theoretical framework: All reviewed studies employed methods consistent with their theoretical rationale. Item 4 examines whether the findings’ interpretation is adequately supported by the data: Most of the papers (*n* = 16) met the criteria. Lastly, Item 5 evaluates the coherence between data collection, analysis, and interpretation: All studies met the criterion. In sum, the overall quality appraisal of the qualitative studies was medium for two studies and high for 16.

*Mixed-methods studies.* Items 1 and 2 probe whether the mixed-methods studies provided a solid rationale for employing this category integrating quantitative and qualitative design appropriately. Item 3 assesses if the overall interpretation based on the integration of both data types was made: all mixed studies (*n* = 4) met this criterion. Item 4 appraises whether the quantitative and qualitative components diverge or not: All mixed studies met this criterion. Lastly, Item 5 evaluates the individual quality of the quantitative and qualitative elements: 3 out of four studies met this criterion. In brief, 3 papers out of 4 reached a high methodological quality appraisal.

### Psychological Constructs of a Sibling’S Experience

The purpose of this section is to summarize the findings for RQ1, i.e.: *What are the main psychological constructs investigated in siblings of persons with disability?*

Findings revealed three main psychological constructs: The sibling relationship (n = 17), sibling-focused parentification (i.e., the caring role that the sibling assumes toward the brother/sister; n = 17), and emotional and behavioral adjustment (i.e., individual’s emotional and behavioral adaptive responses to a stressful situation; n = 14). In a few studies on the population of siblings of persons with disabilities (*n* = 4), well-being was analyzed. Several studies (*n* = 6) examined more than one of the mentioned psychological constructs and were hence included in a hybrid group. Two papers that were conducted during the COVID-19 pandemic were also part of the studies reviewed. Because the stressful environmental conditions related to the pandemic could have affected the way each psychological construct has been experienced by siblings, these papers were reviewed and tabulated separately.

### Impact of Each Investigated Psychological Constructs

This section aims to summarize the findings for RQ2, i.e.: *What is the main role of each psychological construct in siblings of persons with disability experience?*

**Fig. 2:**
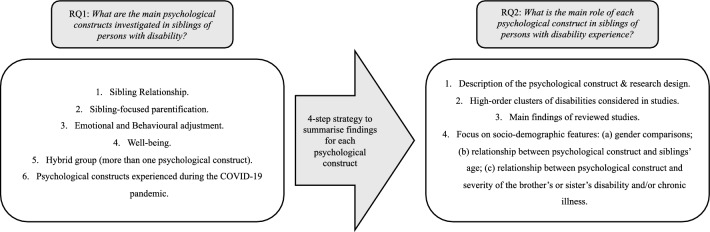
Research questions and main results reached

The impact of each of the psychological constructs identified by answering RQ1 will be summarized by using a four-step strategy. Firstly, a brief description of the psychological construct will be provided, together with the research design (quantitative *vs*. qualitative *vs*. mixed) applied to analyze the construct in each study. Secondly, the high-order clusters of disabilities identified in the reviewed studies will be examined. In addition, the main findings for each psychological construct have been summerized. Finally, the relevant findings according to the socio-demographic features mainly examined in the reviewed studies have been reported. To be accurate, we summed up findings on (a) gender comparisons; (b) the relationship between psychological construct and siblings’ age; and (c) the relationship between psychological construct and severity of the brother’s or sister’s disability and/or chronic illness. Figure 2 shows the strategy.

### Sibling Relationship

Sibling relationships may be the most obvious (Noller, 2005) and enduring kinds of relationships (Milevsky, 2011). They are usually characterized by contradictory feelings, such as love and conflict, affection and rivalry (Buist & Vermande, 2014). The quality of sibling relationships may be affected by the behavior and/or functioning of other family members (Cox & Paley, 1997), such as a child’s brother or sister with a disability and/or a chronic illness.

The electronic search identified 17 studies investigating the quality of sibling relationships between persons with disabilities and their siblings (Avieli et al., 2019; Bhattashali et al., 2018; Braconnier et al., 2018; Corsano et al., 2017; Cuskelly, 2016; Floyd et al., 2016; Guidotti et al., 2021; Hemati Alamdarloo et al., 2021; Noonan et al., 2018; Paul et al., 2022; Rossetti et al., 2020; Shivers, 2019a; Stock et al., 2016; Tomeny et al., 2017a, 2017b; Travers et al., 2020; Tyerman et al., 2019; Yacoub et al., 2018).

Data were collected cross-sectionally in all the studies except one (Floyd et al., 2016), for which they were gathered both cross-sectionally and longitudinally. Seven of the 17 reviewed studies were quantitative (Braconnier et al., 2018; Cuskelly, 2016; Floyd et al., 2016; Hemati Alamdarloo et al., 2021; Shivers, 2019a; Tomeny et al., 2017a, 2017b; Travers et al., 2020), 9 qualitative (Avieli et al., 2019; Bhattashali et al., 2018; Corsano et al., 2017; Noonan et al., 2018; Paul et al., 2022; Rossetti et al., 2020; Stock et al., 2016; Tyerman et al., 2019; Yacoub et al., 2018), and one mixed (Guidotti et al., 2021).

Most of the reviewed studies (*n* = 11) dealt with siblings of persons with mental disorders (Bhattashali et al., 2018; Braconnier et al., 2018; Corsano et al., 2017; Floyd et al., 2016; Guidotti et al., 2021; Noonan et al., 2018; Paul et al., 2022; Shivers, 2019a; Tomeny et al., 2017a, 2017b; Travers et al., 2020; Yacoub et al., 2018). Two studies recruited siblings of people with neurological diseases (Avieli et al., 2019; Tyerman et al., 2019). The other two studies involved siblings of persons with genetic diseases (Cuskelly, 2016; Stock et al., 2016). A study investigated two clusters of disabilities—mental disorders and genetic diseases (Rossetti et al., 2020). The remaining study explored the sibling relationship between TD individuals and their brothers or sisters with sensory diseases (Hemati Alamdarloo et al., 2021).

The reviewed quantitative studies reported mixed findings (Braconnier et al., 2018; Cuskelly, 2016; Floyd et al., 2016; Hemati Alamdarloo et al., 2021; Shivers, 2019a; Tomeny et al., 2017a, 2017b; Travers et al., 2020). On the one hand, several of them showed evidence of good sibling relationships (Braconnier et al., 2018; Cuskelly, 2016; Floyd et al., 2016; Tomeny et al., 2017a, 2017b; Travers et al., 2020). In particular, Travers and colleagues (2020) found that siblings spent more time with their brothers or sisters with disabilities and joined them in recreational activities. A study by Braconnier et al. (2018) revealed that siblings perceived the relationship with their brother or sister with a disability as being more satisfying than the one they had with their parents. Tomeny and colleagues (2017) highlighted that the supportive role played by parents positively affected sibling relationships. Cuskelly’s research (2016) showed that a sibling’s warmth benefited sibling relationships. On the other hand, findings by Hemati Alamdarloo and colleagues (2021) revealed conflictual and poor sibling relationships. Finally, it was shown that both the relationships between persons with disabilities and their siblings and those between TD individuals and their siblings were characterized by the same positive and negative aspects (Shivers, 2019a). The reviewed quantitative studies did not examine gender differences nor the association between the severity of a brother’s or sister’s disability and sibling relationship as a psychological construct. As for the association between the age of the siblings of persons with disabilities and sibling relationship, findings (Floyd et al., 2016) showed that the older the sibling, the better the sibling relationship was.

Findings of qualitative studies (Avieli et al., 2019; Bhattashali et al., 2018; Corsano et al., 2017; Noonan et al., 2018; Paul et al., 2022; Rossetti et al., 2020; Stock et al., 2016; Tyerman et al., 2019; Yacoub et al., 2018) revealed some crucial aspects of sibling relationships. A study by Rossetti and colleagues (2020) showed that the quality of sibling relationships was influenced by the type of a brother’s or sister’s disability. Specific results demonstrated that sibling relationships with brothers or sisters with Down syndrome were characterized by higher levels of reciprocity and involvement in activities compared to sibling relationships with brothers or sisters with autism. Research by Avieli et al. (2019) revealed five sibling relationship patterns: (a) sibling relationships where the sibling was a surrogate parent; (b) sibling relationships characterized by negative feelings, conflict, and rivalry; (c) sibling relationships shaped by ambivalent non-epistemic mental states (i.e., emotions) toward the brother or sister with a disability; (d) sibling relationships where the sibling acted as a mediator in the family; and (e) sibling relationships characterized by closeness and sharing. Several negative factors that may affect the quality of sibling relationships were also identified (Noonan et al., 2018; Paul et al., 2022; Tyerman et al., 2019; Yacoub et al., 2018). These included concern and uncertainty for the future (Corsano et al., 2017; Noonan et al., 2018; Paul et al., 2022), feelings of isolation in the parent-sibling relationship (Stock et al., 2016; Tyerman et al., 2019), difficulty in making friends and sharing one’s situation with others (Corsano et al., 2017), and experience of the social stigma associated with disability (Paul et al., 2022). Furthermore, siblings reported that the pervasive nature of their brother’s or sister’s disability (e.g., autism) negatively affected the quality of the sibling relationship, which was associated with feelings of anxiety, worry, and guilt (Stock et al., 2016; Yacoub et al., 2018).

However, besides an increased sense of responsibility, siblings also reported feelings of love, support, and respect (Bhattashali et al., 2018; Noonan et al., 2018; Paul et al., 2022; Stock et al., 2016; Tyerman et al., 2019; Yacoub et al., 2018), together with a strong sense of personal growth due to their dealing with their brother’s or sister’s disability (Paul et al., 2022). As for gender comparison, a study by Tyerman and colleagues (2019) revealed no significant gender differences in sibling relationships. Findings by Stock et al. (2016) showed that stronger relationships were established between siblings of the same gender. In the other qualitative studies reviewed (Avieli et al., 2019; Bhattashali et al., 2018; Corsano et al., 2017; Noonan et al., 2018; Paul et al., 2022; Rossetti et al., 2020; Yacoub et al., 2018), gender differences were not analyzed. In terms of the association between the age of siblings of persons with disabilities and sibling relationship, Stock et al. (2016) found that the age gap between a sibling and their brother or sister with a disability was directly proportional to their rivalry. To be accurate, considering the developmental stage of the participants' childhood and adolescence (*n* = 6) and youth and adulthood (*n* = 6) have been enrolled. The other studies took into account a wide age range (*n* = 4) and one study does not report this demographic feature. On children and adolescents, studies showed warm and close relationships (Bhattashali et al., 2018; Guidotti et al., 2021) as well as empathy and positive feelings toward their siblings with disabilities (Shivers, 2019a). Nevertheless, these studies reported sporadic family conflicts. On adults, attitudes may depend on the disability. For instance, adult siblings of persons with autism reported fewer positive attitudes compared to those with intellectual disabilities (Rossetti et al., 2020; Tomeny, Ellis et al., 2019), whereas warm and harmonious relationships have been reported by those of persons with Down syndrome (Cuskelly, 2016).

Only one study (Rossetti et al., 2020) investigated the association between sibling relationship and the severity of a brother’s or sister’s disability, with findings revealing that the more pervasive the disorder (e.g., mental disorders), the poorer the sibling relationship was.

Finally, a mixed study by Guidotti and colleagues (2021) found evidence of good sibling relationships. As for gender differences, findings showed that male siblings of persons with disabilities tended to perceive the sibling relationship as being conflictual, while their female counterparts were less inclined to think so. In terms of the association between the age of siblings of persons with disabilities and sibling relationship, findings showed that siblings of adolescents with disabilities were more ashamed, annoyed, and embarrassed than siblings of children with disabilities. The association between sibling relationship and the severity of a brother’s or sister’s disability was not computed in mixed studies.

### Sibling-Focused Parentification

Following Hooper and colleagues (2012), parentification is “*a type of role reversal, boundary distortion, and inverted hierarchy between parents and other family members in which adolescents assume developmentally inappropriate levels of responsibility in the family of origin*” (Hooper et al., 2012; p. 165). The authors argued that this caring role served by the individual can be referred toward the parents (called parent-focused parentification) and/or toward a sibling (called sibling-focused parentification). When a disability occurs, this caring role may be experienced differently by the siblings. For this reason, the review focused on the sibling-focused parentification construct solely.

The electronic search identified 17 studies on the topic (Beffel & Nuttall, 2020; Brady et al., 2019; Brolin et al., 2024; Chiu, 2022; Kale & Siğirtmaç, 2021; Lashewicz, 2018; Eun Lee et al., 2018, 2023; Eun Lee et al., 2019a, 2019b; Eun Lee et al., 2019a, 2019b; Eun Lee et al., 2020; Niedbalski, 2023; Nuttall et al., 2018; Redquest et al., 2020; Rossetti et al., 2018; Tomeny et al., 2016a, 2016b, 2017a, 2017b). Data were collected cross-sectionally in all the studies. In 9 studies (Beffel & Nuttall, 2020; Brolin et al., 2024; Eun Lee et al., 2023; Eun Lee et al., 2019a, 2019b; Eun Lee et al., 2019a, 2019b; Nuttall et al., 2018; Redquest et al., 2020; Tomeny et al., 2016a, 2016b, 2017a, 2017b), quantitative data were gathered, while 6 studies (Brady et al., 2019; Chiu, 2022; Kale & Siğirtmaç, 2021; Lashewicz, 2018; Eun Lee et al., 2018; Niedbalski, 2023), analyzed qualitative data. In 2 studies (Eun Lee et al., 2020; Rossetti et al., 2018), both quantitative and qualitative data were collected.

Most of the reviewed studies examined the experience of siblings of persons with mental disorders. A study by Eun Lee et al., (2019a, 2019b) involved siblings of people with different disabilities: i.e., mental disorders, motor disabilities, and genetic diseases. Research by Kale and Siğirtmaç (2021) focused on siblings of people with genetic diseases and motor disabilities. Finally, the study by Brolin et al. (2024) recruited siblings of people with unspecified disabilities.

The reviewed quantitative studies helped to identify the different roles played by siblings of persons with disabilities. They acted as (a) current and future financial managers (Eun Lee et al., 2019a, 2019b; Redquest et al., 2020), (b) anchors (Eun Lee et al., 2019a, 2019b) or companions (Eun Lee et al., 2019a, 2019b), (c) caregivers (Eun Lee et al., 2019a, 2019b; Tomeny et al., 2016a, 2016b), and (d) facilitators of access to health care services (Eun Lee et al., 2019a, 2019b).

Three studies (Eun Lee et al., 2019a, 2019b; Tomeny et al., 2016a, 2016b, 2017a, 2017b) explored the association between sibling-focused parentification and sibling relationship, revealing conflicting results. More specifically, two studies (Eun Lee et al., 2019a, 2019b; Tomeny et al., 2016a, 2016b) found evidence of good sibling relationships despite the high level of responsibility experienced by the sibling because of parentification. Conversely, a study by Tomeny and colleagues (Tomeny et al., 2017a, 2017b) showed that a high level of responsibility and low perceived social support were associated with poor sibling relationships. Cross-cultural research conducted by Eun Lee et al. (2023) explored sibling-focused parentification in South Korea, Japan, and Taiwan, revealing the siblings’ low engagement in advocacy activities and caregiving. Findings by Beffel et al. (2020) and Nuttall et al. (2018) highlighted the protective role played by the perceived benefits of having a brother or sister with a disability. The authors stated that the siblings’ prosocial behavior (Beffel & Nuttall, 2020) and their future caregiving intentions (Nuttall et al., 2018) were predicted when they perceived their brothers’ or sisters’ disabilities as positively contributing to their own life. Finally, the study by Brolin et al. (2024) found that the caring role was associated with both negative (e.g., mental ill-health, impact on schooling in terms of negative school performance because the caring or being bullied because of the disability of their brother or sister, and a lack of support) and positive outcomes (e.g., increased maturity).

Except for a study by Eun Lee (2023), in which no gender differences were found when exploring sibling-focused parentification as a psychological construct, the other quantitative studies did not examine gender differences.

The age of siblings of persons with disabilities was positively related to parentification (Redquest et al., 2020). In other words, siblings older than 50 years of age showed an increased intensity in caregiving compared to middle-aged (30–49 years) and younger (20–29 years) siblings. One out of 17 reviewed studies recruited children to adolescents, 11 studies considered youth and adulthood, and four studies considered a wide age range. One study did not report the age range of the participants. Together, these studies reported mixed results on children, adolescents, and adults. On the one hand, maturity and personal growth result from the parentification. On the other hand, a low level of general well-being, a negative impact on school performance, and a feeling of lack of support are experiences because related the caring.

Finally, low functioning (Eun Lee et al., 2019a, 2019b), maladaptive behaviors (Eun Lee et al., 2019a, 2019b; Eun Lee et al., 2019a, 2019b), and low level of independence (Eun Lee et al., 2019a, 2019b) shown by brothers/sisters with disabilities were strongly and positively correlated to caregiving demands. The more severe the disability, the higher the level of responsibility perceived by the sibling was.

The reviewed qualitative studies showed that siblings of persons with disabilities mainly played three roles. Firstly, siblings acted as caregivers (Chiu, 2022; Eun Lee et al., 2018; Niedbalski, 2023) on three different levels, being (1) no/minimal caregivers, when they were not or little engaged in intensive caregiving duties; (2) anticipating caregivers, when they stressed the importance of discussing the future of their brother or sister with a disability with their parents; and (3) joint/primary caregivers, when they replaced their parents in caregiving duties. Secondly, findings revealed that siblings also played the role of friend and anchor (Eun Lee et al., 2018), follower (Lashewicz, 2018), and guardian (Brady et al., 2019; Eun Lee et al., 2018) for their brothers or sisters with disabilities. Finally, siblings acted as their brothers’ or sisters’ protectors and advocates within the family (Lashewicz, 2018).

Gender comparisons and the association between sibling-focused parentification and the severity of a brother’s or sister’s disability were not computed in qualitative studies. Only one study (Kale & Siğirtmaç, 2021) argued that parentification occurred regardless of the sibling’s gender. The same study also revealed that older siblings reported having more responsibilities than younger ones. Finally, a study by Chiu (2022) found that siblings—regardless of their age—were willing to help and take care of their brother or sister with a disability.

Two mixed studies were yielded. Both used high-order clustering to investigate the sibling’s role. Research by Eun Lee and colleagues (2020) identified three roles played by siblings of persons with disabilities. These included (1) the non-caregiver, (2) the single caregiver, that is a sibling who takes care of their brother/sister with a disability, and (3) the compound caregiver, a sibling who takes care of both their brother/sister with a disability and another person (e.g., the sibling’s child). A study by Rossetti and colleagues (2018) showed a 5-cluster solution, with the sibling being: (1) a companion, that is, an individual who is a close friend with their brother/sister with a disability; (2) little involved, thus showing low engagement in caregiving; (3) highly involved, when frequently playing the different roles of caregiver, sibling, and advocate; (4) needs-focused, and hence becoming their brother’s or sister’s advocate and informal service coordinator; and (5) professional, that is, their brother’s or sister’s legal representative and advocate. Among the different roles played by siblings of persons with disabilities, those of compound caregivers (Eun Lee et al., 2020) and companions (Rossetti et al., 2018) were the ones associated with a stronger emotional closeness in the sibling relationship.

Gender comparisons, the connection between sibling-focused parentification and the age of siblings of persons with disabilities., and the association between sibling-focused parentification and the severity of a brother’s or sister’s disability were not computed.

### Emotional and Behavioral Adjustment

Adjustment is the individual’s emotional and behavioral adaptive response to a stressful environment or situation and its associated challenges (Harrison & Murray, 2015). Due to the considerable effort required when dealing with disabilities, growing up with a brother or sister with a disability and/or a chronic illness may be compared to living in a stressful environment. Thus, the individual emotional and behavioral adjustment may be impaired.

The electronic search identified 14 cross-sectional studies on the topic (Hanvey et al., 2022; Hayden et al., 2023; Le Boudec et al., 2021; Milevsky & Singer, 2022; O’Neill & Murray, 2016; Orm et al., 2022; Perenc & Pęczkowski, 2018; Shivers, 2019b; Shivers & Kozimor, 2017; Shojaee et al., 2020; Siman-Tov & Adi Sharabi, 2023; Tomeny et al., 2016a, 2016b; Tsai et al., 2016; Yaldız et al., 2021), with no longitudinal research being conducted. Eleven of the 13 cross-sectional reviewed studies were quantitative while the remaining two were qualitative (Hanvey et al., 2022; Milevsky & Singer, 2022). No mixed studies investigating the topic were yielded.

Nine of the eleven quantitative studies included data on siblings of persons with mental disorders (Hayden et al., 2023; O’Neill & Murray, 2016; Shivers, 2019b; Shivers & Kozimor, 2017; Shojaee et al., 2020; Siman-Tov & Adi Sharabi, 2023; Tomeny et al., 2016a, 2016b; Tsai et al., 2016; Yaldız et al., 2021). Two quantitative studies (Orm et al., 2022; Perenc & Pęczkowski, 2018) analyzed more than one high-order cluster of disability (e.g., mental disorders and motor disabilities). One study (Le Boudec et al., 2021) involved siblings of persons with disabilities and/or chronic illnesses whose details were not specified.

The two qualitative studies presented data on siblings of persons with more than one disability, that is, mental disorder and genetic disease (Milevsky & Singer, 2022), and mental disorder and neurological disease (Hanvey et al., 2022).

When analyzing emotional/behavioral adjustment as a psychological construct, the main findings of papers exclusively focusing on emotional adjustment (*n* = 8; Hanvey et al., 2022; Hayden et al., 2023; Milevsky & Singer, 2022; O’Neill & Murray, 2016; Perenc & Pęczkowski, 2018; Shivers, 2019b; Siman-Tov & Adi Sharabi, 2023; Yaldız et al., 2021) and those of studies centered on both emotional and behavioral adjustment (*n* = 6; Boudec et al., 2021; Orm et al., 2022; Shivers & Kozimor, 2017; Shojaee et al., 2020; Tomeny et al., 2016a, 2016b; Tsai et al., 2016) were examined separately. No studies solely investigating behavioral adjustment were identified.

### Emotional Adjustment

Studies exclusively focused on emotional adjustment revealed that the siblings’ main responses to a stressful situation caused by their brothers’ or sisters’ disabilities were guilt, depressive symptoms, anxiety, stress, and empathy (Hanvey et al., 2022; Hayden et al., 2023; Milevsky & Singer, 2022; O’Neill & Murray, 2016; Perenc & Pęczkowski, 2018; Shivers, 2019b). More than 50% of the siblings of persons with disabilities involved in a study (Shivers, 2019b) reported having experienced feelings of guilt, described their sibling relationships as being poor, showed a high level of depressive symptoms and a low level of well-being. However, siblings of people with disabilities who had developed some independent living abilities experienced lower levels of mental distress showed a higher level of well-being and had a better quality of life (Hayden et al., 2023). Depressive symptoms and anxiety were assessed by comparing the experience of siblings of persons with different types of disabilities (O’Neill & Murray, 2016; Siman-Tov & Adi Sharabi, 2023). To be accurate, in the study by O’Neill and Murray (2016) siblings of persons with autism and Prader-Willi syndrome reported higher levels of anxiety than siblings of TD individuals. Siblings of individuals with autism and developmental disorders of unknown etiology showed higher levels of depression than the siblings of TD people. No significant differences in depressive symptoms and anxiety were found between siblings of persons with Down syndrome and siblings of TD individuals. The study by Siman-Tov & Adi Sharabi (2023) compared siblings of brother or sister with autism and intellectual disabilities revealing that the first showed less distress than the latter.

Although the negative impact of disability was reported in most of the reviewed studies, high levels of empathy were also identified in several of them (Milevsky & Singer, 2022; Perenc & Pęczkowski, 2018). Higher levels of cognitive and emotional empathy were reported by siblings of persons with motor disabilities than by siblings of TD individuals (Perenc & Pęczkowski, 2018). Kindness, patience, acceptance of others (Milevsky & Singer, 2022), and more attention to the other’s wants and needs (Yaldız et al., 2021) were also part of the positive aspects reported in the reviewed studies.

In terms of gender differences, most of the reviewed studies exclusively focused on emotional adjustment revealed that female siblings of persons with disabilities showed higher levels of positive and negative emotional symptoms compared to their male counterparts. Both females with a brother/sister with a disability and those of a TD individual reported higher levels of anxiety than male siblings (O’Neill & Murray, 2016; Yaldız et al., 2021). Furthermore, females experienced stronger feelings of guilt than their male counterparts (Shivers, 2019b). Conversely, two studies found that female siblings of persons with disabilities showed a higher level of cognitive and emotional empathy (Perenc & Pęczkowski, 2018), involvement, self-efficacy, and well-being (Siman-Tov & Adi Sharabi, 2023) than males. Only one study (Siman-Tov & Adi Sharabi, 2023) reported that male siblings of persons with disabilities experienced more loneliness than female counterparts.

As for the association between the age of the siblings of persons with disabilities and emotional adjustment as a psychological construct, the findings showed mixed results. On the one hand, the siblings’ age was positively correlated to cognitive empathy (Perenc & Pęczkowski, 2018): the older the sibling, the higher the level of cognitive empathy was. On the other hand, the study by O’Neill and Murray (2016) outlined that younger sibling of persons with genetic diseases (i.e., Down syndrome or Prader-Willi syndrome) showed high levels of anxiety (in the case of Down syndrome) and depressive symptoms (in the case of Prader-Willi syndrome). Furthermore, the authors found that the elder siblings of persons with developmental disorders of unknown etiology showed increased levels of anxiety. Considering children and adolescents of person with disability, they reported high levels of prosocial behaviors suggesting earlier and greater resilience and social engagement. On adults, results showed that less distress and high well-being depending on the level of the independence of their brother or sister with disability.

Finally, the association between the psychological construct and the severity of the brother’s or sister’s disability showed some interesting results. The low level of independence and functioning of the brother/sister with a disability was inversely proportional to the TD sibling’s feelings of guilt (Shivers, 2019b) and levels of emotional symptoms and distress (Hayden et al., 2023), while being directly proportional to the level of the sibling’s well-being (Hayden et al., 2023).

### Emotional and Behavioral Adjustment

In the studies focused on both emotional and behavioral adjustment (Le Boudec et al., 2021; Orm et al., 2022; Shivers & Kozimor, 2017; Shojaee et al., 2020; Tomeny et al., 2016a, 2016b; Tsai et al., 2016), the main internalizing/externalizing symptoms detected were hyperactivity, hostility, anxiety, somatic and emotional problems. The levels of hyperactivity of siblings of persons with mental disorders (i.e., autism, intellectual and developmental disorders) were higher than those shown by siblings of TD individuals. Furthermore, the siblings of persons with autism showed more emotional/internalizing symptoms than the siblings of TD individuals (Shojaee et al., 2020). Additionally, siblings without disabilities reported more difficulties in behavioral adjustment in terms of the prosocial domain (Tsai et al., 2016). Siblings of persons with single disabilities reported less hostility and anxiety than siblings of persons with co-occurring disabilities (e.g., intellectual disability and depression or anxiety) (Shivers & Kozimor, 2017). Finally, a study by Orm (2022) found that the siblings of persons with mental disorders or physical disabilities showed higher levels of prosocial behavior than the siblings of TD individuals.

As for gender comparison, findings revealed that female siblings of persons with disabilities reported more somatic problems than their male counterparts, while male siblings of persons with disabilities showed more aggressive behaviors than siblings of TD individuals (Le Boudec et al., 2021). The analysis of the association between the psychological construct and the siblings’ age showed no significant results.

Finally, the severity of the brother’s or sister’s disability was negatively associated with their sibling’s emotional and behavioral adjustment (Tomeny et al., 2016a, 2016b): the more severe the disability was, the higher the levels of maladjustment reported by siblings were.

### Well-being

Well-being is a multidimensional psychological construct covering a broad spectrum of domains, including physical, emotional, mental, social, and behavioral components (WHOQoL Group, 1993). When disability occurs in the family, it may not be surprising that this construct may be affected.

The electronic search conducted identified four papers on the topic (Correia & Seabra-Santos, 2022; Hallion et al., 2018; Koukouriki & Soulis, 2020; Lahaije et al., 2023). Three of them analyzed quantitative data (Hallion et al., 2018; Koukouriki & Soulis, 2020; Lahaije et al., 2023), while the remaining one was a qualitative study (Correia & Seabra-Santos, 2022).

In terms of high-order clustering inventoried for the present review purposes (see Methodological Characteristics of the Reviewed Studies section), a study by Hallion and colleagues (2018) involved siblings of persons with chronic illnesses and disabilities whose details were not specified. The other three studies (Correia & Seabra-Santos, 2022; Koukouriki & Soulis, 2020; Lahaije et al., 2023) analyzed the experience of siblings of persons with mental disorders.

Hallion and colleagues (2018) found no differences in the psychological well-being of siblings of persons with disabilities and siblings of TD individuals. A study by Koukouriki et & Soulis (2020) showed that siblings of persons with autism reported lower levels of well-being compared to siblings of TD individuals. Finally, the study by Lahaije and colleagues (2023) revealed that siblings of persons with disabilities perceived higher levels of emotional and material well-being than their parents. A qualitative study by Correia & Seabra-Santos (2022) revealed that the well-being of siblings of persons with disabilities was both positively and negatively impacted by their brother’s or sister’s disability. On the one hand, siblings stated that having a brother/sister with a disability influenced their personal growth, in terms of maturity, tolerance, understanding, patience, and strength. On the other hand, siblings acknowledged the difficulties and limitations imposed by disability.

No gender comparisons were analyzed when investigating well-being. The association between their age and well-being was computed only by Lahaije and colleagues (2023). Nevertheless, the authors included both parents and siblings of persons with disabilities in the analysis. Consequently, a more in-depth sub-analysis according to age range of participants is not possible. The impact of the severity of the disability on siblings’ well-being has not been examined.

### Hybrid Studies

Studies that analyzed more than one of the specific psychological constructs identified in RQ1 were clustered as hybrid studies. Six papers met this criterion (Cebula et al., 2019; Jones et al., 2019; Kulisch et al., 2024; Levante et al., 2023a, 2023b, 2023c; Shivers et al., 2019; Zaidman-Zait et al., 2020). Four of them investigated both sibling relationship and emotional/behavioral adjustment, and one of them (Levante et al., 2023a, 2023b, 2023c) focused on sibling-focused parentification, social support, and parent-sibling relationships. The remaining one (Kulisch et al., 2024) investigated both behavioral adjustment and well-being.

In all the hybrid studies reviewed, quantitative data were collected cross-sectionally. Two studies (Jones et al., 2019; Zaidman-Zait et al., 2020) exclusively involved siblings of individuals with mental disorders. Other two studies analyzed more than one disability, with one investigating mental disorders and genetic diseases (Shivers et al., 2019), and the other dealing with genetic diseases, motor disabilities, sensory diseases, and mental disorders (both as a single and co-occurring disability) (Levante et al., 2023a, 2023b, 2023c). One study focused on siblings of persons with genetic diseases (Cebula et al., 2019). Finally, the remaining study (Kulisch et al., 2024) involved siblings of persons with unspecified disabilities.

A study by Shivers and colleagues (2019) found that siblings of persons with autism reported higher levels of emotional and behavioral maladjustment and less warmth in the sibling relationship compared to siblings of individuals with Down syndrome. Furthermore, the levels of emotional and behavioral adjustment reported by siblings of persons with disabilities were similar to those shown by the general population (Cebula et al., 2019). A study by Jones et al. (2019) revealed that siblings showed low levels of emotional and behavioral adjustment when their brother/sister with a disability reported high levels of such problems. Findings by Zaidman-Zait et al. (2020) showed higher levels of closeness and lower levels of conflict in relationships between persons with disabilities and their siblings compared to relationships between TD siblings. Furthermore, Zaidman-Zait et al. (2020) also found that good sibling relationships were associated with the highest levels of emotional and behavioral adjustment. A study by Levante and colleagues (Levante et al., 2023a, 2023b, 2023c) showed that the sibling’s high levels of distress and a negative parent-sibling relationship detrimentally affected the interplay between sibling-focused parentification and sibling relationship. The same study revealed that the benefits of parentification and the social support perceived by the siblings of persons with disabilities contributed to reducing their levels of distress. Finally, the study by Kulisch et al. (2024) revealed that coping strategies (in terms of minimization, situation control, positive self-instructions, and the need for social support) were positively associated with quality of life.

None of the reviewed studies analyzed gender differences nor the association between psychological constructs and the severity of the disability. Only one study (Levante et al., 2023a, 2023b, 2023c) included gender as a covariate in the mediation model, with no differences in terms of gender being found. As for the association between the age of siblings of persons with disabilities and the psychological constructs investigated, on children and adolescents Cebula et al. (2019) found that the older the sibling, the less warm the relationship was.

In terms of hybrid studies, three of them explore the experiences of children and adolescents. The study by Zaidman-Zait et al. (2020) found that siblings of children with disabilities show higher levels of empathy, less conflict and rivalry, and greater support and investment in the sibling relationship than siblings of typically developing (TD) individuals.

### The Experience of Siblings of Persons with Disabilities during COVID-19

Although the COVID-19 pandemic heavily affected the functioning of individuals and several studies investigated the impact of restrictive measures on vulnerable populations (Asquini et al., 2021; Corbett et al., 2021; Levante et al., 2019, 2022; Rosencrans et al., 2021), the electronic search conducted identified only two studies (Dorsman et al., 2023; Redquest et al., 2021) focused on the sibling population. These were cross-sectional and mixed studies that aimed to explore the experience of siblings of persons with mental disorders during the pandemic lockdowns. In particular, the study by Redquest et al. (2021) investigated the support given to brothers/sisters with disabilities, the emotional adjustment, and the quality of family relationships. Data were collected from two groups: siblings of individuals with mental disorders who lived in the same house as their brothers or sisters, and siblings of individuals with mental disorders who did not live together with their brothers or sisters. In sum, results showed that all the siblings involved in the study constantly supported their brothers or sisters with disabilities, regardless of where they lived. Similarly, all of the siblings recruited reported being concerned for the health and well-being of their brothers or sisters with disabilities. The siblings who lived with their brothers or sisters with disabilities stated that the increased amount of time spent with them positively affected their sibling relationship.

The second study (Dorsman et al., 2023) carried out during the pandemic explored the roles served by siblings of persons with disabilities and the impact of pandemic-related restrictive measures on sibling relationship. Results outlined that the most assumed roles by siblings were brother/sister and legal representative/administrator/mentor. In addition, siblings of persons with disabilities reported that the social distancing imposed by the pandemic negatively affected the sibling relationship.

For both studies, gender comparisons were not computed, and the association between psychological constructs and the severity of the brother’s or sister’s disability was not analyzed. Finally, on youths and adults no association between psychological constructs and the age of the siblings of persons with disabilities was found.

## Discussion

This systematic integrative review aimed to contribute to summarizing the empirical literature on the developing field of research on the psychological impact of a brother’s or sister’s disability on the functioning of their TD sibling. Therefore, following a PEO format, two research questions were formulated: “*What are the main psychological constructs investigated in siblings of persons with disability?*” (RQ1), and “*What is the main role of each psychological construct in siblings of persons with disability experience*” (RQ2). Sixty studies met the inclusion criteria based on the PICOS protocol and were included in the systematic review. Their methodological quality was appraised and showed that most of the reviewed studies reported a high (*n* = 45) quality; 15 reached a medium quality. The main characteristics and findings were tabulated and summarized narratively.

To identify the strengths and limitations of the reviewed studies, an overview of their methodological characteristics was provided. The findings prompted further considerations.

As most of the reviewed studies were conducted in Global North countries, future studies should involve the sibling population in Global South countries: cross-cultural studies may help to understand how having a brother or sister with a disability may impact the sibling’s experience. In addition, exploring the WEIRD and non-WEIRD populations could be an interesting and novel field of research. Not only would this lead to better identifying the psychological constructs that play a pivotal role in each specific culture, but it may also provide information to design culture-specific intervention programs.

A further issue to consider is related to the design of the reviewed studies, as data were collected cross-sectionally in most of them. Despite being challenging, gathering data longitudinally might help to understand how psychological constructs change over time. Similarly, conducting a meta-analysis combining relevant data might provide evidence of the impact of disability on each psychological construct.

Moreover, just under a fifth of the reviewed studies (*n* = 11) compared siblings of people with disabilities to siblings of TD individuals. A more comprehensive analysis might lead to identifying the psychological constructs that are most heavily affected by disability.

As for the sample recruited in the reviewed studies, two main issues should be highlighted. The first issue concerns gender distribution, as a descriptive analysis revealed that most of the studies recruited an unbalanced sample in terms of the gender of the siblings of persons with disabilities. This methodological flaw prevented an appropriate interpretation and generalization of the data. The second issue to consider is the wide age range of the siblings of persons with disabilities involved in the studies, a flaw that may be caused by difficulty in recruiting siblings at a specific developmental stage. Again, findings should be interpreted cautiously. Indeed, each developmental stage involves age-specific tasks (Erikson, 1994), based on which psychological constructs should be investigated. Consequently, future studies should solve such issues to provide clinical guidance in designing gender- and developmental stage-specific intervention programs.

Finally, the review of the selected studies showed that the most investigated cluster of disability was that of mental disorders. Although the pervasive nature of mental disorders (Hastings & Pet al.,as, 2014; Mayes et al., 2012) might significantly impact the psychological constructs of a sibling’s well-being (Shojaee et al., 2020; Tomeny et al., 2016a, 2016b), it would also be useful to investigate other clusters of disabilities, considering their different levels of severity.

The first research question addressed by this systematic review aimed to identify the main psychological constructs of a sibling’s experience investigated in the selected studies. Findings revealed that the main psychological constructs analyzed were sibling relationship (*n* = 17), sibling-focused parentification (*n* = 17), and emotional/behavioral adjustment (*n* = 14). Only four of the reviewed studies focused on the well-being of siblings of persons with disabilities. The studies investigating more than one psychological construct were included in a hybrid group (*n* = 6). Finally, two studies were identified that explored the role of siblings of persons with disabilities, their emotional adjustment, and the quality of family relationships during the COVID-19 lockdowns. Findings suggest that future studies should extend knowledge of the most investigated psychological constructs and inform intervention programs. Additional research should aim at exploring topics that are often overlooked and their potential impact on the functioning of siblings of persons with disabilities.

An analysis of the findings for the second research question, which aimed at summarizing the main role of each identified psychological constructs, has led to making several suggestions.

The main psychological construct investigated among the sibling population was sibling relationship. In the reviewed studies, quantitative, qualitative, and mixed data were collected cross-sectionally. However, longitudinal studies would allow one to examine the magnitude of the change occurring in sibling relationships over time. As the main cluster of disability investigated was that of mental disorders, other clusters should be considered to extend knowledge of the topic. The reviewed studies yielded inconsistent results. On one side, they provided evidence of good, warm, and satisfying sibling relationships characterized by siblings spending time with their brothers or sisters with disability and participating in various activities. On the other side, findings showed conflictual and negative sibling relationships characterized by ambivalent feelings and rivalry.

The second most examined psychological construct was sibling-focused parentification, which was investigated by collecting quantitative, qualitative, and mixed data on siblings of persons with mental disorders. Therefore, longitudinal studies should also be designed, and other clusters of disabilities should be considered. The reviewed studies showed that siblings of persons with disabilities mainly acted as caregivers, with feelings of warmth and closeness being reported, together with a sense of duty toward other family members such as parents. The association between sibling relationship and sibling-focused parentification turned out to be the most examined in the literature. Nevertheless, findings were inconsistent, which suggests that such an interplay should be better analyzed in future longitudinal investigations.

The emotional and behavioral adjustment of siblings of persons with disabilities was the third most investigated psychological construct. As the reviewed studies analyzed quantitative and qualitative data on siblings of persons with mental disorders, it would be advisable to conduct some longitudinal and mixed studies in the future. Findings revealed that anxiety/depression, guilt, empathy, aggressive behaviors, and conduct problems were the most emotional/internalizing and behavioral/externalizing symptoms reported by siblings of persons with disabilities.

As for the studies investigating the well-being of siblings of persons with disabilities, the review findings highlighted that quantitative and qualitative data were collected cross-sectionally. Therefore, mixed and longitudinal studies should also be conducted in the future. The need for more in-depth investigations on the well-being of the siblings of persons with disabilities has been confirmed by both the inconsistent findings of the reviewed research and the low number of studies carried out on the topic. This psychological construct may play a crucial role in the development of intervention programs. Indeed, following the Complete Mental Health model proposed by Keyes (2002), the enhancement of individual well-being is essential to promote human flourishing, in terms of high levels of well-being and the absence of mental illness. Consequently, such a construct should be investigated in more detail.

Finally, all of the studies included in the hybrid cluster were cross-sectional and quantitative. For this reason, qualitative and/or mixed longitudinal studies should also be designed in the future. As for the psychological constructs analyzed, the reviewed studies focused on sibling relationship and the emotional and behavioral adjustment of siblings of persons with mental disorders.

Although this systematic review included papers published during the COVID-19 pandemic, only two of the studies selected focused on the issues faced by the sibling population in that period. Due to the short- and long-term effects that a pandemic may have on vulnerable populations and any individuals’ mental health (Alonso-Esteban et al., 2021; Bianco et al., 2021; Colizzi et al., 2020; Levante et al., 2019, 2022, 2024, 2023a, 2023b, 2023c; Panchal et al., 2021; Petrocchi et al., 2020; Quarta et al., 2022, 2023), further research should be conducted on the matter. The issues to explore should include the changes in the relationship between siblings of persons with disabilities owing to pandemic lockdowns and people being forced to live together, the potential change in the siblings’ role and/or in the perception of their responsibilities, the development and use of strategies to cope with problems arising when dealing with brothers or sisters with disabilities, and caregiving in a period in which it was not possible to access health care services.

The analysis of each psychological construct has led to the identification of three cross-cutting issues that are relevant to each of the constructs. In terms of the gender of the siblings of persons with disabilities, findings showed that females reported stronger feelings of guilt and higher levels of both anxiety and depression compared to their male counterparts. Furthermore, female siblings felt a stronger sense of responsibility than male siblings. Female siblings showed higher levels of empathy and better sibling relationships than male siblings. As for the siblings’ well-being, no gender comparisons were computed. It is important to point out that such results should be interpreted cautiously. When considering the association between the age of the siblings and the investigated psychological constructs, it should be mentioned that the age range of the siblings of persons with disabilities involved in the studies was often wide, which prevented an accurate interpretation of findings. Such an issue will be better discussed in the Future Recommendations section. Finally, the review showed that the severity of a brother’s or sister’s disability significantly impacted the psychological functioning of their sibling. In other words, the higher level of severity of a brother’s or sister’s disability resulted in a poor sibling relationship, more responsibilities in caregiving, and high levels of emotional and behavioral maladjustment. However, these results should be interpreted cautiously, since the main cluster of disability considered was that of mental disorders.

### Implications for Practitioners

The findings of this systematic integrative review may play a role in clinical settings, as health professionals could take them into account when designing intervention programs aimed at siblings of persons with disabilities. These interventions may become instrumental in improving the psychological functioning of siblings in several ways. Specifically, our results may put the foundation for suggesting a model nesting the main psychological constructs extracted by the current systematic integrative review. Among them (i.e., sibling relationship, sibling-focused parentification, emotional/behavioral adjustment, and well-being), sibling-focused parentification may be the key psychological construct of target intervention. It may serve as a direct predictive factor of the quality of sibling relationships which, in turn, may have a cascade effect on the sibling’s psychological well-being and emotional/behavioral adjustment. In this vein, sibling relationships may be a simultaneous outcome and mediator. In other words, intervention programs may support the siblings in their caregiver role by mitigating the emotional burden related to the responsibilities and duties because of caregiving for their brother or sister with disabilities. Consequently, low levels of sibling-focused parentification may promote a positive attitude toward their brother/sister with a disability, an optimistic perspective toward the future, and the overall siblings’ well-being and emotional/behavioral adjustment. Therefore, the sibling relationship may directly benefit from low levels of sibling-focused parentification. Also, the sibling relationship may mediate the path between sibling-focused parentification, well-being, and emotional/behavioral adjustment: The lower responsibilities and duties because of the disability of their brother/sister (sibling-focused parentification), the high well-being and emotional/behavioral adjustment via the mediation of a high-quality sibling relationship.

Albeit the suggested model pieces together the main psychological constructs investigated in the sibling population, an additional innovative construct could moderate the suggested relationship between sibling-focused parentification and sibling relationships. Considering the scarce literature on the acceptance of disability as a resource in sibling populations than other ones (i.e., parents; Lecciso et al., 2013a, 2013b), the suggested model may pave the way for adding this construct in further investigations. For instance, the acceptance of disability may be conceived as a personal resource that arranges the sibling to face caregiving demands positively affecting the sibling relationship. Last, but not least exploring the role of sociodemographic features—i.e., siblings’ gender and age—and of the type of disability in the suggested model. Additionally, may be key to test the model considering the cultural background of the target sibling population. It is worth highlighting that although each relationship suggested in the model is supported by a sound theoretical framework, it has to be tested cross-culturally and in a gender-balanced sample.

### Future Research and Intervention Recommendations

The systematic integrative review carried out has led to providing a series of recommendations for future research and intervention on the vulnerable population analyzed in the selected studies.

On the research field, it would be interesting to understand whether a sibling’s culture may influence how they are psychologically affected by having a brother or sister with a disability. As the systematic review showed, most of the studies were carried out in Global North countries, while only a few investigated the sibling population in Global South countries. In particular, one of the psychological constructs identified in this systematic review was the role played by siblings of persons with disabilities, which may also be analyzed in terms of cultural aspects (Rossetti & Hall, 2015). More in-depth investigations may help to address different research questions, such as “Are there any differences in the caregiving roles played by siblings of persons with disabilities in different cultures?”, and/or “How does parental education impact the siblings’ perception of their caregiving responsibilities toward their brothers or sisters with disabilities?”.

A second issue concerns the characteristics of the sample recruited in the studies, in terms of (1) the groups of participants, (2) the gender of the siblings of persons with disabilities, and (3) the age of the siblings of persons with disabilities. As for the groups of participants involved in the studies, descriptive results showed that most of the analyses explored psychological constructs only in the experimental group including siblings of persons with disabilities. This did not help to understand what psychological construct(s) was(were) the most affected by the brother’s or sister’s disability. Therefore, future investigations could compare the functioning of the siblings of persons with disabilities to that of siblings of TD individuals. Furthermore, as our review showed that studies have mostly focused on siblings of people with mental disorders, further research is needed on other clusters of disabilities (e.g., motor disability, sensory disability). This will allow a more comprehensive understanding of siblings' experiences in different disability contexts.

As for the gender of the siblings of persons with disabilities, the systematic review showed that about 82% of the studies involved more female than male participants. The Social Role Theory (Eagly, 2009; Pinho & Gaunt, 2024) helps to read this result. According to authors, societal expectations and norms shape female behaviors: women learn that they are the main family care providers, who manage home duties, and take care to each family member, especially when disability occurs earliest. Albeit vicious, this trend is supported by the high prevalence of females in participating in studies. More studies on male siblings are required to deepen their perspective and experiences regarding the disability of their brother and sister.

Furthermore, it would be interesting to investigate gender differences regarding the siblings’ culture. Investigating gender differences in sibling roles across cultures would provide deeper insights into how these roles are culturally constructed and the impact they have on family dynamics and individual well-being.

Finally, when considering the characteristics of the sample recruited in the selected studies, the age range of the siblings of persons with disabilities was wide, which led to interpreting findings cautiously. According to Erikson’s theory of psychosocial development (1994), each age range is characterized by specific tasks and goals, which should be considered when exploring psychological dimensions. For instance, the types of roles served by siblings of persons with disabilities could change if they are children (whom developmental tasks are to play and discover the social world), adolescents (who work for a sense of belonging to a group of peers and for the development of one’s identity), or adults (who engage in decision-making processes about their future, as moving out, pursuing a career, and that of their brothers or sisters with disabilities). The way to experience the impact of disability has a cascade effect on the quality of sibling relationship, emotional/behavioral adjustment, and well-being as well. Therefore, future studies should focus on specific age ranges, and/or samples should be clustered according to life-cycle tasks (Arnett, 2000).

Another aspect that should be considered in future investigations is related to the study design (longitudinal *vs*. cross-sectional) and the types of data collected (quantitative *vs*. qualitative *vs*. mixed). As the review showed, no longitudinal studies were carried out, which meant that causality relationships between constructs could not be identified. Although cross-sectional results provided general information about the psychological impact of disabilities on the functioning of siblings, the direction of the relationship between variables could not be determined.

A final issue concerns the strategies to be used in future studies. In several of the reviewed studies, strategies were specifically developed for each study, and/or no standardized qualitative strategies were applied. Therefore, results had to be interpreted cautiously. Future studies should aim at extending the range of tools used in this field of research to interpret the findings more accurately.

Findings summarize the psychological constructs mainly investigated by scholars, and which can be considered in individual and systemic as well intervention programs. For instance, they could be focused on the care burden experienced by siblings to develop and/or promote adaptive coping strategies. In turn, sibling could be trained to use these strategies to match their caring responsibilities without carelessness of their personal and professional needs and interests. Individual well-being could benefit from this. Additionally, family dynamics could be gained from sibling-focused interventions. For instance, the pivotal role served by the trust in promoting interpersonal relationships (Petrocchi et al., 2021) and theory of mind ability (Rotenberg et al., 2015) has been demonstrated. The essential role of these competencies is undoubted. Thus, intervention promoting this trait (i.e., interpersonal trust) could improve not only the quality of the parent-sibling relationship but also the sibling relationship. Additionally, the quality of sibling relationship and the individual well-being may benefit from interventions promoting the siblings’ acceptance of their brother or sister diagnosis. To our knowledge, trust in sibling relationship has been poorly investigated (Persram et al., 2022) and no studies on siblings’ reaction to diagnosis of disability has been carried out; thus, much more investigations are needed.

### Strengths and Limitations

The main strength of this systematic review lies in its focus on how siblings are psychologically affected by their brothers’ or sisters’ disability, regardless of the clusters of disabilities identified in the selected studies. Such an approach has helped to explore all the psychological constructs investigated in the empirical literature.

The limitations of this systematic review are connected with the studies selected. Several studies involved more female than male participants. In several studies, the participants’ age range was too wide. In other cases, the sample size was not adequate. All these limitations restrict the transferability and generalization of the findings of this review.
