# Correction: Siblings of Persons with Disabilities: A Systematic Integrative Review of the Empirical Literature

**DOI:** 10.1007/s10567-024-00510-6

**Published:** 2024-11-29

**Authors:** Annalisa Levante, Chiara Martis, Cristina Maria Del Prete, Paola Martino, Patrizia Primiceri, Flavia Lecciso

**Affiliations:** 1 Department of Human and Social Sciences, University of Salento, Via di Valesio, 73100 Lecce, Italy; 2 Lab of Applied Psychology, Department of Human and Social Sciences, University of Salento, Via di Valesio, 73100 Lecce, Italy; 3 District of Rehabilitation, Local Health Service, 73100, P.zza Bottazzi, Lecce, Italy; 4 Office for Inclusion of Individuals with Disability, University of Salento, Via di Valesio, 73100 Lecce, Italy

**Correction to: Clinical Child and Family Psychology Review** 10.1007/s10567-024-00502-6

The original version of this article unfortunately contained an error in text reference citation.

The reference Kulisch et al. 2024, was cited in the text incorrectly with different spelling in each occurrences such as Kulish et al., Koulish et al., Kulisch et al., but the correct citation is Kulisch et al., 2024.

The original article has been corrected.

